# Synthesis of triethoxysilylated cyclen derivatives, grafting on magnetic mesoporous silica nanoparticles and application to metal ion adsorption†

**DOI:** 10.1039/d1ra01581h

**Published:** 2021-03-12

**Authors:** Hao Li, Mathilde Ménard, Ani Vardanyan, Clarence Charnay, Laurence Raehm, Erwan Oliviero, Gulaim A. Seisenbaeva, Roser Pleixats, Jean-Olivier Durand

**Affiliations:** ICGM, Univ. Montpellier, CNRS, ENSCM Case 1701, Place Eugène Bataillon, CEDEX 05 34095 Montpellier France jean-olivier.durand@umontpellier.fr; Department of Chemistry and Centro de Innovación en Química Avanzada (ORFEO-CINQA), Universitat Autònoma de Barcelona 08193-Cerdanyola del Vallès Barcelona Spain Roser.Pleixats@uab.cat; Department of Molecular Sciences, Swedish University of Agricultural Sciences 750 07 Uppsala Sweden Gulaim.Seisenbaeva@slu.se

## Abstract

The synthesis through click chemistry of triethoxysilylated cyclen derivative-based ligands is described. Different methods were used such as the copper catalyzed Huisgen's reaction, or thiol–ene reaction for the functionalization of the cyclen scaffold with azidopropyltriethoxysilane or mercaptopropyltriethoxysilane, respectively. These ligands were then grafted on magnetic mesoporous silica nanoparticles (MMSN) for extraction and separation of Ni(ii) and Co(ii) metal ions from model solutions. The bare and ligand-modified MMSN materials revealed high adsorption capacity (1.0–2.13 mmol g^−1^) and quick adsorption kinetics, achieving over 80% of the total capacity in 1–2 hours.

## Abstract 2

The adsorption of metal ions through ligand-functionalized magnetic mesoporous silica nanoparticles is described.

## Graphical abstract

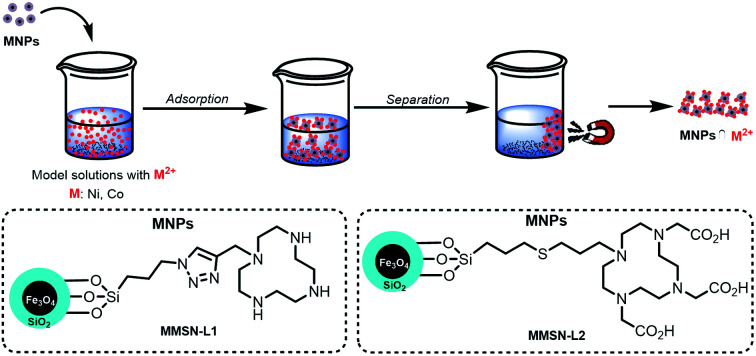

## Introduction

The anchoring of cyclen derivatives on silica-based materials has attracted attention over the last two decades. The main application concerns magnetic resonance imaging^1–9^ or radiolabelling^10,11^ with metal-DOTA-functionalized silica-based nanoparticles for theranostic applications, but not only. Other applications relate to ESR studies with Cu(ii),^12^ or Cu(ii) catalysis,^13^ Pd(ii) recovery,^14^ or photoresponsive materials with Eu(iii) or Tb(iii).^15,16^ Therefore applications of silica-based materials functionalized with cyclens are very diverse, promising, and present a high interest. Two ways are used for the functionalization of silica materials with cyclen derivatives. The first one consists of anchoring a reactive function such as a chloro or amino group on silica using silanes such as chloro or aminopropyltriethoxysilane and then to react this function with an activated acid of DOTA derivative for aminopropyltriethoxysilane or with an amino of cyclen derivative for chloropropyltriethoxysilane. The drawback of this strategy is that the coupling reaction is usually not quantitative and the remaining reactive function on the surface of the material is not completely functionalized. The second possibility consists in preparing triethoxysilylated cyclen derivatives and then coupling the derivatives on the surface of the materials. The preparation involves the coupling reaction of silanes (iodopropyltriethoxysilane, aminopropyltriethoxysilane) with cyclen or activated DOTA derivatives.

Magnetic mesoporous silica nanoparticles (MMSN) have gained much attention the last decade for bio-application, sensing or catalysis and the field has been comprehensively reviewed.^17–22^ In the course of our studies on functionalized MMSN for metal complexation,^23^ we were interested in the preparation of triethoxysilylated cyclen-based ligands, through click Cu-catalyzed azide–alkyne Huisgen's reaction^24^ with azidopropyltriethoxysilane or click thiol–ene reaction^25^ with mercaptopropyltriethoxysilane, which have not been investigated for this purpose. The cyclen derivatives were then grafted on the surface of MMSN and the materials were used for the extraction and separation of Ni(ii) and Co(ii) ions.

## Experimental

### Materials and methods

Cyclen (CAS 294-90-6, Fluorochem), di-*tert*-butyl dicarbonate (CAS 24424-99-5, Sigma Aldrich), allyl bromide (CAS 106-95-6, Sigma Aldrich), propargyl bromide (CAS 106-96-7, Fluorochem), (3-mercaptopropyl)triethoxysilane (CAS 4420-74-0, Sigma Aldrich), (3-azidopropyl)triethoxysilane (CAS 83315-69-9, Abcr GmbH), iron oxide (CAS 20344-49-4, Sigma Aldrich), oleic acid (CAS 112-80-1, Sigma Aldrich), *n*-docosane (CAS 629-97-0, Acros), oleylamine (CAS 112-90-3, Acros), tetraethyl orthosilicate TEOS (CAS 78-10-4, Sigma Aldrich), cetyltrimethylammonium bromide CTAB (CAS 57-09-0, Sigma Aldrich), pentane (CAS 109-66-0, Fisher), chloroform (CAS 67-66-3, Sigma Aldrich), trifluoroacetic acid (CAS 76-05-1, Fluorochem), triethylamine (CAS 121-44-8, Merck), dichloromethane (CAS 76-09-2, Carlo Erba), ethyl acetate (CAS 141-78-6, VWR), ethanol and acetone (Honeywell).

FTIR spectra were recorded in the 4000–400 cm^−1^ range using 32 scans at a nominal resolution of 4 cm^−1^ with a Perkin Elmer 100 FT spectrophotometer equipped with an ATR unit. TEM images were recorded with JEOL 1200 EXII microscope and an JEOL 2200 FS (JEOL Europe SAS, Croissy Sur Seine, France). For the purpose of TEM analysis, the sample particles were dispersed in ethanol and then dropped onto copper grids covered with porous carbon films. TGA analyses were performed with a thermal analyser STA 409 Lux® (Netzsch) in the range 25–800 °C by a heating speed of 5 °C min^−1^.

### Preparation of MMSN

#### Synthesis of magnetic iron oxide nanoparticles (MIONs)

The MIONs were synthesized by thermal decomposition according to a previous reported protocol.^26^ Briefly, 0.18 g of hydrated iron oxide(ii) were mixed with 5 g of docosane and 3.2 g of oleic acid. The mixture was first stirred under vacuum for 30 min and then placed under Ar flow for another 30 min. Then the reaction occurred at 340 °C for 1.5 h. After completion of the reaction the MIONs were washed and recovered by three successive centrifugations at 20 krpm for 10 min and redispersion by ultrasounds for few minutes: (1) by adding 15 mL of pentane and 30 mL of a mixture of ether : ethanol (2 : 1, v/v) to the as-synthesized product; (2) by adding 2 mL of pentane and 30 mL of a mixture of ether : ethanol (2 : 1, v/v) to the collected black product; (3) by adding 30 mL of a mixture of ether : ethanol (1 : 1, v/v) to the collected black product. Finally, the MIONs were stored in 15 mL of chloroform and stabilized by adding 200 μL of oleylamine.

#### Synthesis of MMSN^27^

2 g of CTAB were first dissolved in 250 mL of distilled water for 1 h at 70 °C under stirring. The surfactant solution was then cooled down to 40 °C and 5 mL from the previous synthesized MIONs dispersed in chloroform were added. The emulsion was first put under vigorous stirring without heating for 30 min and then the temperature was risen to 70 °C and kept for additional 40 min under stirring. Hence, the phase transfer of MIONs succeeded, 300 mL of distilled water slightly basified by 300 μL of 2 M NaOH was added to the solution under stirring. When the solution reached 60 °C, 7.5 mL of TEOS followed by 12.5 mL of EtOAc were quickly added to the mixture. Then the condensation reaction was conducted for 3 h at 70 °C under stirring. The final MMSN were recovered by centrifugation, concentrated to 100 mL and washed two times: twice with water and twice with EtOH at 95%. After each centrifugation step the MMSN were redispersed in the appropriate washing solvent under sonication for few minutes. To extract the CTAB the MMSN were soaked twice in a solution of ammonium nitrate (6 g L^−1^ in EtOH 90%) and put under stirring at 70 °C for 2 h. After each extraction, the washing steps by centrifugation described above in water and ethanol were performed. Finally, the MMSN were redispersed in 25 mL of EtOH 96%. IR *ν* (ATR) (cm^−1^): 2979.6, 1067.8, 963.6, 797.2, 451.3. TGA (air, 5 °C min^−1^, 20–800 °C) loss mass 14.0%. Zeta potential: *ζ* = −32 mV, pH = 7.24. DLS: 183 nm.

### Synthesis of ligand

#### Synthesis of tri-*tert*-butyl 10-((1-(3-(triethoxysilyl)propyl)-1*H*-1,2,3-triazol-4-yl)methyl)-1,4,7,10-tetraazacyclododecane-1,4,7-tricarboxylate, L1-Boc

To a dry, 100 mL Schlenk flask equipped with a stir bar and under argon atmosphere, tri-*tert*-butyl 10-(prop-2-yn-1-yl)-1,4,7,10-tetraazacyclododecane-1,4,7-tricarboxylate 1 (763.5 mg, 1.50 mmol), CuBr(PPh_3_)_3_ (69.5 mg, 0.075 mmol) and anhydrous THF (15 mL) were added. Then, (3-azidopropyl)triethoxysilane (380.9 mg, 1.54 mmol) was added by using a syringe. The resulting mixture was stirred at 60 °C (argon atmosphere) for 48 h. Then, the solvent was evaporated under reduced pressure and the crude product was obtained as colorless solid (1.16 g, 100% yield) without any purification. ^1^H NMR (360 MHz, CDCl_3_) *δ* (ppm): 7.69–7.64 (m, 1H), 4.32 (t, *J* = 7.2 Hz, 2H), 3.90 (s, 2H), 3.82 (q, *J* = 7.2 Hz, 6H), 3.55 (s, 4H), 3.36 (br s, 8H), 2.80–2.62 (m, 4H), 2.04–1.96 (m, 2H), 1.46–1.43 (m, 27H), 1.21 (t, *J* = 7.2 Hz, 9H), 0.58 (t, *J* = 7.2 Hz, 2H) (Fig. S1†). IR *ν* (ATR) (cm^−1^): 2971.9, 2926.3, 1682.4, 1457.9, 1391.0, 1153.8, 1074.3, 943.0, 643.0. MS (ESI) *m*/*z*: 279.1, 473.3, 721.4, 730.5, 758.5; HRMS (ESI) *m*/*z* [M + H]^+^ calcd for C_35_H_67_N_7_O_9_Si: 758.4842, found: 758.4835.

#### Synthesis of tri-*tert*-butyl-2,2′,2′′-(10-(3-((3-(triethoxysilyl)propyl)thio)propyl)-1,4,7,10-tetraazacyclododecane-1,4,7-triyl)triacetate, L2-*^t^*Bu

In a 50 mL Schlenk tube under nitrogen, tri-*tert*-butyl-2,2′,2′′-(10-allyl-1,4,7,10-tetraazacyclododecane-1,4,7-triyl)triacetate 4 (554.8 mg, 2.06 mmol) and 2,2-dimethoxy-1,2-diphenylethanone (105.3 mg, 0.41 mmol) were dissolved in anhydrous THF (10.0 mL). Then (3-mercaptopropyl)triethoxysilane (489.9 g, 2.06 mmol) was added and the stirred mixture was irradiated with a UV lamp at 365 nm under argon atmosphere for 72 h. Then, the solvent was removed under reduced pressure and the desired product L2-*^t^*Bu was obtained as yellow oil (1.62 g, 99% yield). ^1^H NMR (360 MHz, CDCl_3_) *δ* (ppm): 3.93–3.79 (m, 6H), 3.77–3.73 (m, 2H), 3.55–3.00 (m, 8H), 2.82–2.54 (m, 18H), 1.87–1.86 (m, 2H), 1.85–1.71 (m, 2H), 1.46 (s, 27H), 1.25–1.21 (m, 9H), 0.74 (t, *J* = 7.2 Hz, 2H) (Fig. S2†). IR *ν* (ATR) (cm^−1^): 2974.3, 1721.8, 1454.6, 1366.6, 1251.0, 1147.1, 1102.9, 956.8, 782.8, 474.5. MS (ESI) *m*/*z*: 403.3, 459.3, 486.3, 515.4, 516.4, 534.4, 555.4, 621.4, 661.5, 793.5; HRMS (ESI) *m*/*z* [M]^+^ calcd for C_38_H_76_N_4_O_9_SSi: 793.5175, found: 793.5150.

#### Synthesis of tri-*tert*-butyl 10-((1-(3-(triethoxysilyl)propyl)-1*H*-1,2,3-triazol-4-yl)methyl)-1,4,7,10-tetraazacyclododecane-1,4,7-triacetate, L3-*^t^*Bu

In a 10 mL microwave reaction tube, tri-*tert*-butyl 2,2′,2′′-(10-(prop-2-yn-1-yl)-1,4,7,10-tetraazacyclododecane-1,4,7-triyl)triacetate 5 (50 mg, 0.09 mmol) CuBr(PPh_3_)_3_ (11 mg, 0.012 mmol) and (3-azidopropyl)triethoxysilane (30 mg, 0.12 mmol) were added. Then all reactants were dissolved in 3 mL of anhydrous THF. The mixture was stirred under microwaves at 100 °C (200 Wmax) for 4 cycles of 10 min. After completion of the reaction, the solvent was removed under reduced pressure and the desired product L3-*^t^*Bu was obtained. ^1^H NMR (200 MHz, CDCl_3_) *δ* (ppm): 7.49–7.45 (m, 1H), 4.33 (t, *J* = 8 Hz, 2H), 3.84–3.74 (m, 6H), 3.77–3.73 (m, 2H), 3.42–3.21 (m, 6H), 2.91–2.82 (m, 12H), 2.60 (br s, 4H), 2.00–1.83 (m, 2H), 1.43 (s, 27H), 1.23–1.11 (m, 9H), 0.60 (t, *J* = 8 Hz, 2H); ^13^C NMR (126 MHz, CDCl_3_) *δ* (ppm): 171, 146, 123, 81, 58, 57, 56, 55, 54, 52, 29, 25, 19, 8; ^29^Si NMR (99 MHz, CDCl_3_) *δ* (ppm): −46, (Fig. S3†). MS (ESI) *m*/*z*: 800.5, 553.4, 279.1, 170.1, 98.5; HRMS (ESI) *m*/*z* [M + H]^+^ calcd for C_38_H_74_N_7_O_9_Si: 800.5239, found: 800.5312.

### Preparation of MMSN grafted with L*n*-Boc/*^t^*Bu

In a 50 mL round-bottom flask equipped with a Dean–Stark apparatus, ligand L*n*-Boc/*^t^*Bu (0.1 mmol), MMSN (100 mg), H_2_O (500 μL) and Et_3_N (25 μL) were sonicated for 2 h, and then the mixture was heated in DMF (10 mL) at 120 °C for 24 h. After this time, the suspension was centrifugated (20 000 rpm at 25 °C for 15 min). The solid was washed successively with H_2_O (2 × 30 mL), ethanol (2 × 30 mL) and acetone (2 × 30 mL) (sonication for 10 min, 10 min for centrifugation), dried under vacuum, and finally crushed to give the grafted material MMSN-L*n*-Boc/*^t^*Bu as black solid.

#### MMSN-L1-Boc

IR *ν* (ATR) (cm^−1^): 2925.2, 2854.4, 1657.8, 1060.2, 966.4, 796.2, 551.0, 446.8. TGA (air, 5 °C min^−1^, 20–800 °C) loss mass 15.8%. Zeta potential: *ζ* = −6 mV, pH = 6.85. DLS: 216 nm.

#### MMSN-L2-*^t^*Bu

IR *ν* (ATR) (cm^−1^): 2924.9, 2852.8, 1656.6, 1062.8, 967.2, 797.1, 555.9, 444.2. TGA (air, 5 °C min^−1^, 20–800 °C) loss mass 15.8%. Zeta potential: *ζ* = −14 mV, pH = 6.75. DLS: 237 nm.

### Preparation of MMSN-L*n*

The MMSN-L*n*-Boc/*^t^*Bu were dissolved in TFA/CH_2_Cl_2_ (5 : 1) solution, the mixture was stirred at room temperature for 60 minutes. Then the NPs were collected by centrifugation (20 000 rpm at 25 °C for 10 min). The material MMSN-L1 was washed successively with EtOH (2 × 30 mL), saturated NaHCO_3_ solution (2 × 30 mL), distilled water (2 × 30 mL), acetone (2 × 30 mL) and EtOH (2 × 30 mL). The material MMSN-L2 was washed successively with EtOH (2 × 30 mL), acetone (2 × 30 mL) and EtOH (2 × 30 mL). Finally, both MMSN-L*n*-were dried under vacuum for several hours.

#### MMSN-L1

IR *ν* (ATR) (cm^−1^): 2925.4, 2852.8, 1656.2, 1081.3, 966.9, 797.3, 553.8, 447.5. TGA (air, 5 °C min^−1^, 20–800 °C) loss mass 15.3%. Zeta potential: *ζ* = 39 mV, pH = 6.99. Elemental analysis: 7.33% C, 1.63% H, 1.46% N.

#### MMSN-L2

IR *ν* (ATR) (cm^−1^): 2925.1, 2855.4, 1658.0, 1063.4, 964.7, 797.4, 554.8, 450.7. TGA (air, 5 °C min^−1^, 20–800 °C) loss mass 17.8%. Zeta potential: *ζ* = −36 mV, pH = 6.65. Elemental analysis: 9.05% C, 1.84% H, 1.01% N, 1.76% S.

For isotherm experiments stock solutions of Ni^2+^ and Co^2+^ (50 mM) were prepared using nitrate salts and the final concentrations were adjusted by dilution with Milli-Q water (varying between 0.5–25 mM). Samples (10 mg) of magnetic nanoparticles (MNPs) were mixed with 10 mL of metal cation solution in plastic tubes of 50 mL and put on a shaker for 24 hours. After each experiment, MNPs were centrifuged (7000*g*) for 10 min and an aliquot (1 mL) was separated to determine the metal concentration in the remaining solution. The samples were first diluted 5–10 times (depending on the initial metal concentration) and titrated afterwards with EDTA using xylenol orange as an indicator. For each sample, the titrations were repeated 3 times, and the average concentration was calculated.

The uptake of metal cation by the magnetic nanoparticles was calculated according to the equation:Up = (*C*_0_ − *C*_e_) × *V*/*m*where *C*_0_ is the initial metal concentration, which was also measured by titration; *C*_e_ is the equilibrium metal concentration; *V* is the metal solution volume which was kept constant; *m* is the NPs weight.

For kinetic tests, the magnetic nanoadsorbents (40 mg) were mixed with 40 mL of metal cation solutions (with 10 mM initial metal concentration) and the uptake was measured after set interval of times. For that purpose, an aliquot of 1 mL was separated, diluted 10 times and the remaining metal concentration was evaluated by titration with EDTA and xylenol orange.

For selectivity test, mixed metal solution was prepared with final concentration of 5 mM + 5 mM Ni and Co ions. The overall concentration was measured by titration with EDTA and xylenol orange as an indicator. For adsorption experiment, 20 mg magnetic nanoparticles were mixed with 20 mL metal solution in 50 mL falcon tubes, and the tubes were put on a shaker for 24 hours. After 24 hours, the nanoparticles were separated from metal solution by centrifugation (7000*g*) and the remaining metal content was measured by titration (Table 2). The magnetic nanoparticles were dried under nitrogen atmosphere and the adsorbed metal distribution was determined by EDS analysis.

For the study of stability and reuse, 30 mg of each nanoparticle samples were mixed in 30 mL metal solution with 10 mM metal ion concentration for 24 hours. Afterwards, the solutions with nanoparticles were centrifuged (7000 rpm) and the metal content was estimated by titrations with EDTA and xylenol orange as an indicator. The desorption was carried out by adding 30 mL nitric acid (1 mM) and put on a shaker for 24 hours. Nanoparticles were centrifuged for 15 min (7000 rpm) and the amount of desorbed metal ions was estimated by titrations with EDTA. Afterwards, the nanoparticle samples were washed with MilliQ water and second round of adsorption experiment was carried out, with the same metal ion concentration and volume. Adsorption and desorption experiments were repeated 3 times.

## Results and discussion

**Scheme 1:**
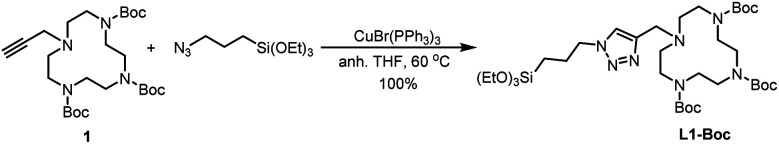
Synthesis of L1-Boc.

Ligand L1-Boc was synthesized in quantitative yield by a copper-catalyzed alkyne–azide cycloaddition reaction (CuAAC) of the alkyne 1 ^28,29^ with commercial (3-azidopropyl)triethoxysilane under anhydrous conditions,^30^ using CuBr(PPh_3_)_3_ as catalyst in dry THF at 60 °C (Scheme 1).

**Scheme 2:**
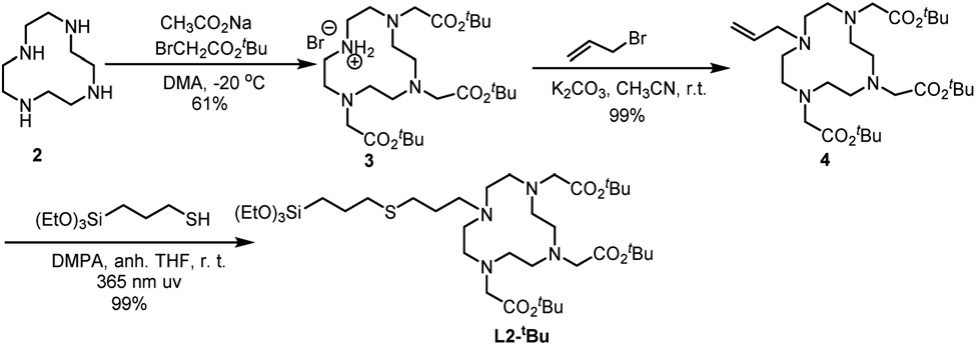
Synthesis of L2-*^t^*Bu.

Ligand L2-*^t^*Bu was obtained through a thiol–alkene click reaction between the suitable alkene and 3-mercaptopropyltriethoxysilane. The synthesis of L2-*^t^*Bu is summarized in Scheme 2. The commercial cyclen 2 was treated with three equivalents of *tert*-butyl 2-bromoacetate in the presence of sodium acetate to afford compound 3,^31^ which was reacted with allyl bromide in acetonitrile at room temperature in the presence of potassium carbonate to give 4 in an overall 60% yield from 2. The alkene 4 and (3-mercaptopropyl)triethoxysilane were irradiated at 365 nm using 2,2-dimethoxy-1,2-diphenylethanone (DMPA) as initiator, and the desired ligand L2-*^t^*Bu was obtained in quantitative yield.

**Scheme 3:**
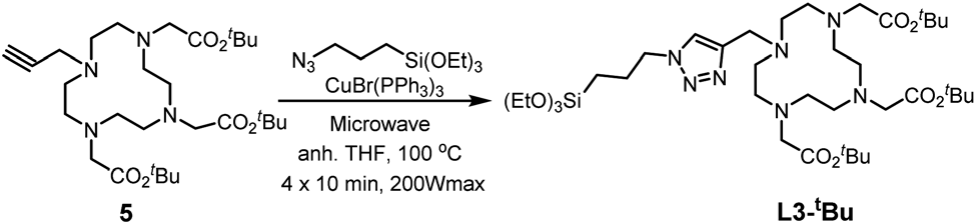
Microwave assisted synthesis of L3-*^t^*Bu.

Through these two synthesis routes, two new cyclen-derivatives that are, to the best of our knowledge, not described in the literature, were successfully synthesized. Nevertheless, these syntheses, although successful, have reaction times lasting several days. Thus, a modification of the synthesis procedure was explored to synthesize L1-Boc by click chemistry under microwaves, which allows to obtain the molecule in less than 1 h *versus* 48 hours through standard protocol. Briefly, precursor 1, the silylated azide and 6 mol% of catalyst were dissolved in dry THF and put in the microwaves at 100 °C, 200 Wmax, for 3 cycles of 10 min. The obtaining of the right molecule was assessed through HNMR analysis. Since the microwave approach was successful to synthesize L1-Boc the protocol was adapted for the synthesis of the derivative named L3-*^t^*Bu, which could not be obtained through standard approaches. Thereby precursor 5 (see ESI† for the synthesis of 5),^32^ the silylated azide and 12 mol% of catalyst were dissolved in dry THF and stirred under microwaves at 100 °C (200 Wmax) for 4 cycles of 10 min (Scheme 3). This new cyclen derivative was then identified by ^1^H NMR, ^13^C NMR, ^29^Si NMR and HRMS. During the experiments, to set up the best protocols for microwave assisted synthesis of L1-Boc and L3-*^t^*Bu two key parameters were identified: the catalyst amount and the number of 10 min cycles. Indeed, it was observed that with less catalyst the reaction did not occur fully and a mixture of starting and expected molecules was obtained. In addition, increasing the reaction time (6 cycles of 10 min for example) tended to damage the molecules: the ^1^H NMR signal at 2.63 ppm (4*H*) for L1-Boc and at 2.59 ppm (4*H*) for L3-*^t^*Bu representative of the cyclen were missing.

Thus, after establishing the best protocol, microwaves allowed the efficient synthesis of L1-Boc and L3-*^t^*Bu and represented a promising approach toward simple, cheaper and faster cyclen derivatives synthesis.

**Fig. 1:**
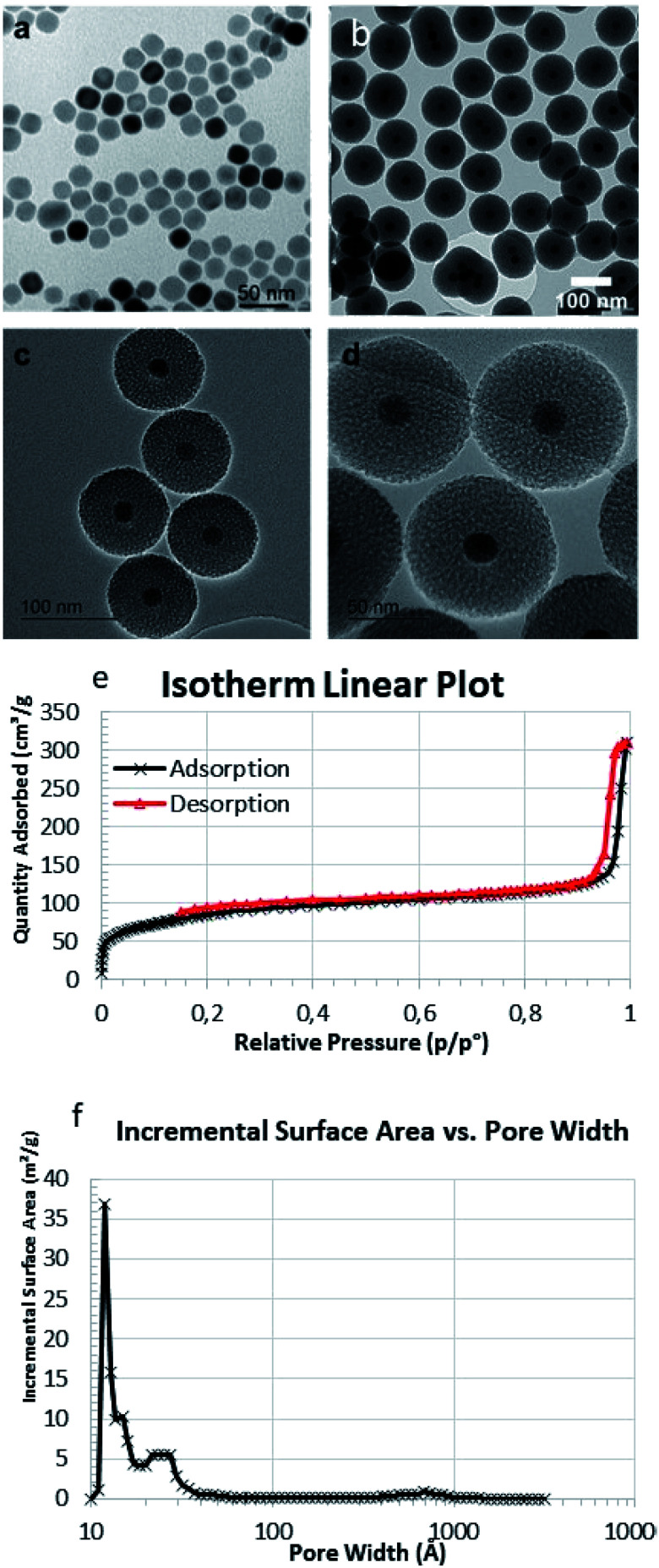
TEM image of (a) MIONs and (b) MMSN, (c and d) HRTEM images of MMSN showing the mesoporosity. (e and f) N_2_ adsorption–desorption analysis (BET) and pore diameters (DFT) of MMSN.

Core–shell nanoparticles were synthesised using ∼20 nm iron oxide cores obtained through thermal decomposition. A silica mesoporous shell was then grown around the core. These nanoparticles exhibited an overall diameter of 102 ± 4 nm by TEM images (Fig. 1a and b), and HRTEM showed the mesoporosity (Fig. 1c and d). The mesoporosity was further confirmed with N_2_ adsorption–desorption (BET) (Fig. 1e and f). MMSN showed type I–IV isotherm characteristic of micro–mesoporous materials. The specific surface area was 302 m^2^ g^−1^ and pore diameters (DFT method) showed two populations at 1.3 nm (microporosity) and a larger pic at 2.5 nm (mesoporosity). TEM was in agreement with the average hydrodynamic diameter (183 nm) of the nanoparticles determined by DLS (Fig. S4a†). These nanoparticles were also analyzed by zeta potential, TGA and IR (Fig. S4b–S6†).

**Fig. 2:**
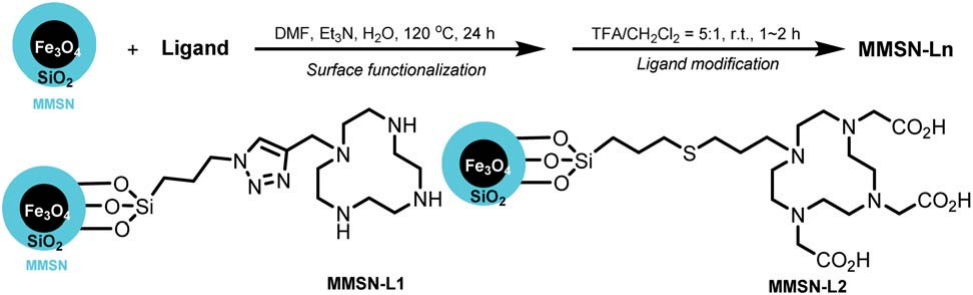
Cyclen functionalized magnetic mesoporous core–shell silica nanoparticles (MMSN).

Then ligands (L1-Boc and L2-*^t^*Bu) were used to modify the surface of MMSN by post-grafting (Fig. 2). We initially investigated different grafting conditions with ligand L2-*^t^*Bu, and the reaction was monitored by FTIR (γC=O 1657 cm^−1^) (see Table S1 and Fig. S7 in ESI†). In refluxing toluene no reaction occurred. The best conditions were established as follows: in DMF at 120 °C with 2 mmol L2-*^t^*Bu per g MMSN, using water and Et_3_N as catalysts. The same procedure was used with L1-Boc. The BOC and *t*-Bu groups were then classically cleaved using TFA to lead to MMSN-L1 and MMSN-L2, respectively. To further assess and estimate the surface functionalisation the nanoparticles were characterised by DLS (Fig. S8†), zeta potential (Fig. S9†), FTIR (Fig. S10 and S11†), TGA (Fig. S12†), and elemental analysis. The data are summarised in Table 1.

**Table 1:** Characterisation of raw *versus* surface functionalized MMSN

Sample	DLS^a^ (nm)	pH	Zeta potential (mV)	EA^b^ (mmol g^−1^)	TGA^c^ (wt%)
MMSN	183	7.24	−32	—	Reference
MMSN-L1-Boc	216	6.85	−6	nd^d^	5.31 wt%
MMSN-L1	nd	6.99	39	0.15	4.34 wt%
MMSN-L2-*^t^*Bu	237	6.75	−14	nd	7.76 wt%
MMSN-L2	nd	6.65	−36	0.18	7.19 wt%

a Hydrodynamic diameters from DLS.

b EA: elemental analysis, experimental value of N content.

c Determined by mass loss with TGA (a heating rate of 5 °C min^−1^ from 30 to 800 °C).

d Not determined.

DLS showed a slight increase of the hydrodynamic diameters from MMSN to MMSN-L1-Boc and MSN-L2-*^t^*Bu in agreement with a successful grafting of the ligand on the surface of MMSN (Fig. S8†). This also indicates that the NPs keep their colloidal stability in water even after their surface modification. The grafting was also confirmed by zeta potential measurements performed around neutral pH 7. From raw NPs to grafted NPs, the zeta-potential increased due to the presence of organic coating (Fig. S9†). After TFA treatment, zeta potential became highly positive with MMSN-L1 in agreement with the presence of ammoniums at neutral pH whereas a strong decrease was noticed with MMSN-L2 as carboxylate groups were deprotected. The high zeta-potential positive value for MMSN-L1 and negative one for MMSN-L2 also indicate a good colloidal stability of these nano-objects at a neutral pH. Finally, the grafting content was determined by TGA analysis (Fig. S11†) and elemental analysis (Table 1). TGA analysis showed a grafting of 4.34 wt% for MMSN-L1 which corresponds to a grafting of 0.12 mmol g^−1^ and 7.19 wt% for MMSN-L2 which corresponds to a grafting of 0.15 mmol g^−1^. These results were confirmed by elemental analysis on N which indicated a grafting of 0.15 and 0.18 mmol g^−1^ for MMSN-L1 and MMSN-L2, respectively.

The prepared magnetic core–shell silica nanoparticles were studied for the adsorption of Ni^2+^ and Co^2+^ from aqueous solutions at room temperature.

**Fig. 3:**
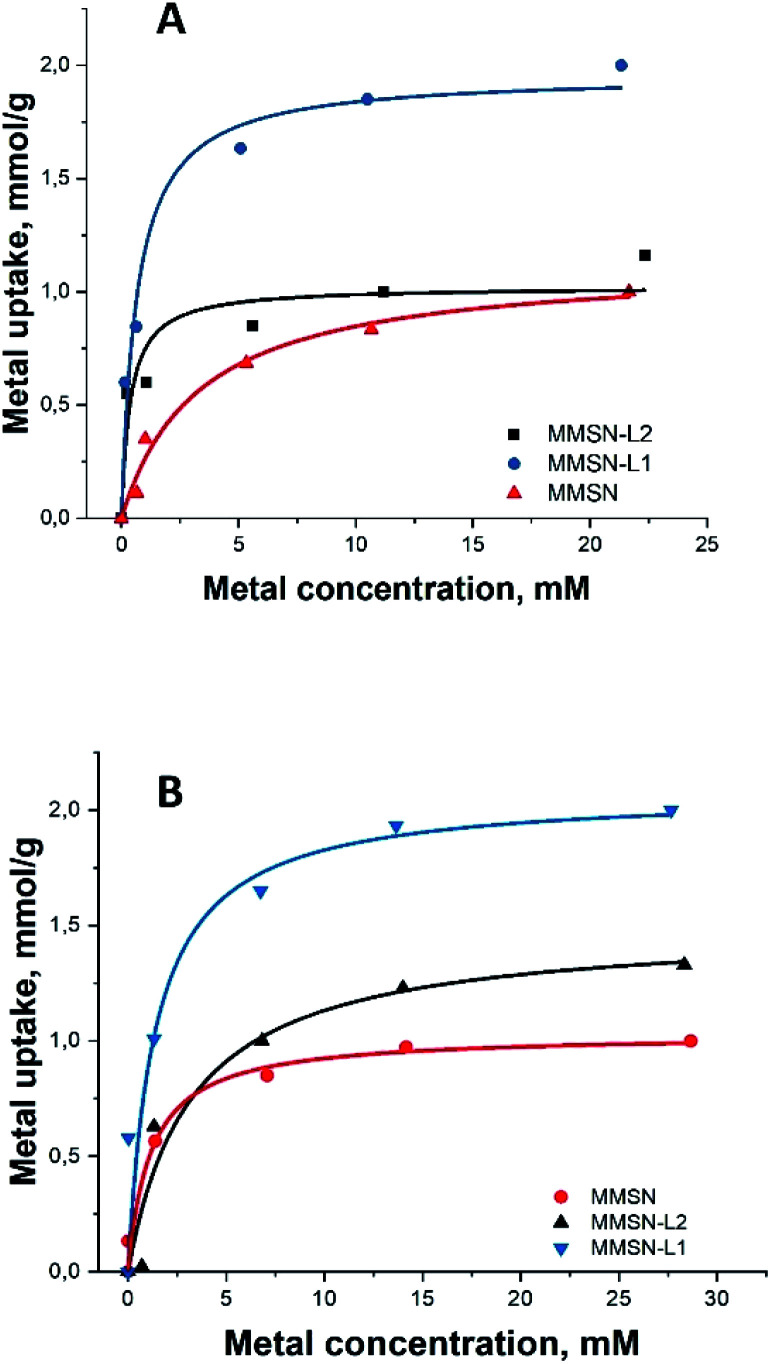
Metal adsorption isotherms: (A and B) Langmuir isotherms for Ni^2+^ and Co^2+^, respectively (for Freundlich isotherms for Ni^2+^ and Co^2+^, respectively, please, see ESI Fig. S13†).

All the magnetic nanoparticles showed similar maximum adsorption capacity (varying between 1–2.13 mmol g^−1^), MMSN having the lowest capacity for both heavy metals (Table 2). For both heavy metals, adsorption isotherms were best fit with Langmuir curves model (Fig. 3), having higher correlation coefficients compared to Freundlich (Fig. S13†), indicating similar mechanisms with uniform binding sites in a monolayer adsorption.

**Table 2:** Average maximum adsorption capacity for each magnetic nanoadsorbent and heavy metal (mmol g^−1^)

Entry	Sample	Ni	Co	Ni : Co = 1 : 1
1	MMSN	1.00	1.00	1.01
2	MMSN-L1	2.13	2.00	0.997
3	MMSN-L2	1.16	1.33	0.94

**Fig. 4:**
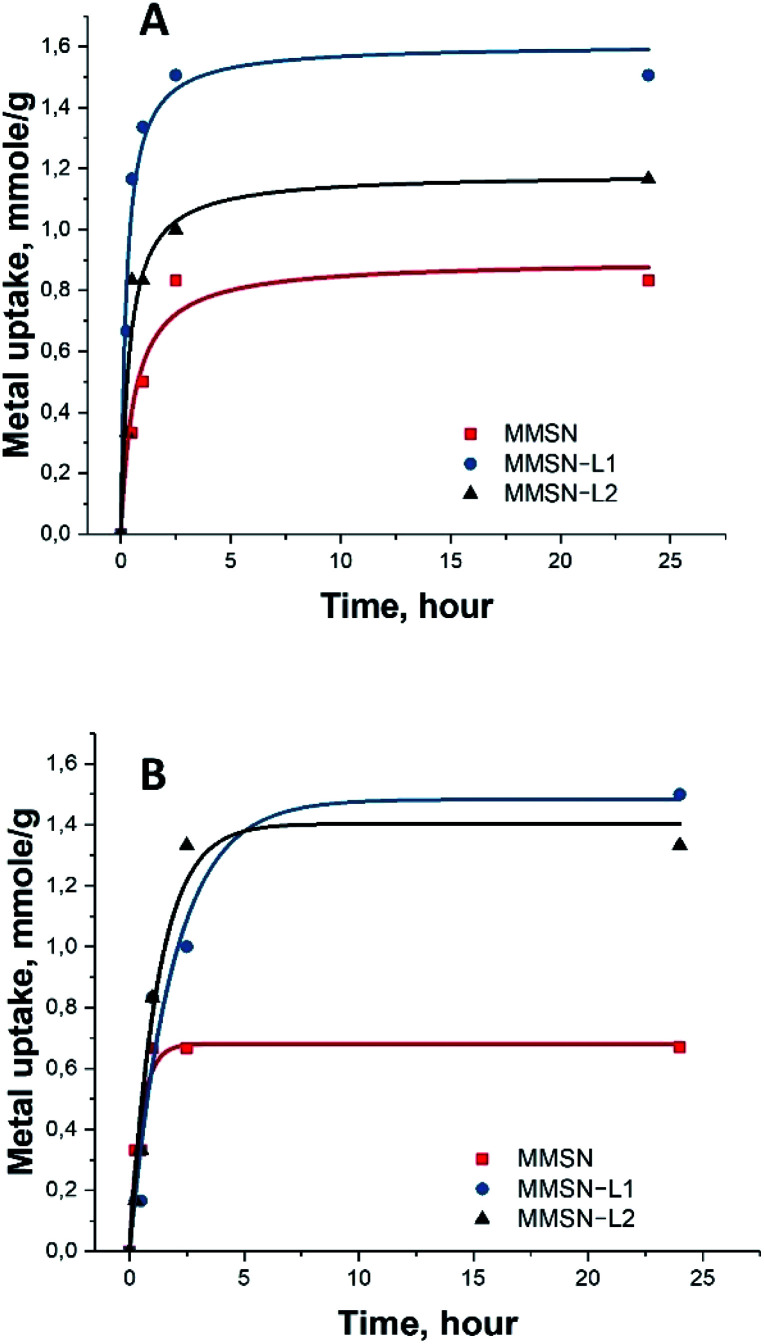
Adsorption kinetics of two heavy metals for (A) nickel(ii), (B) cobalt(ii).

Note that the measurements for the isotherm and the kinetics were carried out under different conditions. Final concentrations in the measurements of isotherms were considerably higher (25 mM) along with lower amount of the applied adsorbent (10 mg) to achieve saturation (Fig. 3). In case of kinetic measurements the solution was much less concentrated (10 mM), while the amount of adsorbent was, in contrast, higher in order to distinguish the first steps in adsorption. The saturation of the adsorbent was not achieved in the kinetic measurements (Fig. 4).

The kinetic test results showed fast uptake at the first 1–2 hours of metal interaction with magnetic nanoparticles, reaching over 80% of the total capacity. Slower uptake was found after these 2 hours and the equilibrium was reached after 5 hours (Fig. 4). This indicated quick and unhindered diffusion through the mesopores.

It is interesting to note that bare, not surface modified MMSN display high capacity in uptake of both Ni(ii) and Co(ii) cations, most probably due to their very high negative surface charge as manifested by strong zeta-potential (−32 mV). Particles modified by L2, acting presumably as a predominantly hard base in Pearson classification, display almost the same capacity as bare silica. This can be explained by some consumption and shielding of the silicate functions after grafting of the cyclen functions, which is compensated by the carboxylate ones in reactivity. On the contrary, the MMSN modified by L1, a soft base in Pearson classification reveal strongly enhanced adsorption characteristics, indicating that the ligand is acting for heavy metal cations as an azo-crown functionality forming stable surface complexes with soft Pearson acids such as Ni(ii) and Co(ii).

From the results of the selectivity tests with Ni : Co = 1 : 1 mixture it is apparent that magnetic nanoparticles have lower adsorption capacity when introduced to mixed metal solution. This could be explained by possible competition for the adsorption sites between the two metal ions, making it more difficult for the ligand to bind the metals. Ni^2+^ and Co^2+^ cations can possess different coordination geometry when binding to soft ligands in Pearson classification. Ni^2+^ cations are prone to Jahn–Teller distortion while Co^2+^ usually display undistorted octahedral coordination. On the contrary, MMSN nanoparticles without any ligands revealed similar results with previous experiments (with single metals only), showing that there is no competition between Ni and Co without any ligands attached to nanoparticles.

EDS analysis gave similar results in regards with selectivity of magnetic nanoparticles (see ESI, Tables S2–S5†). The numbers for L2 and MMSN for contents of both metals are rather similar and in general relatively low, being approximately twice higher for L1. This ratio is reflecting the data on total capacity of the materials (see Fig. 3). This indicates that the enhanced surface of silica itself makes also a strong contribution to adsorption along with complexation to ligands.

Recyclability of the materials was studied, testing the uptake and release capacity in 4 consecutive adsorption–desorption cycles. The results, summarised in Fig. S14,† show unaltered functional characteristics for ligand-free reference material MMSN and functionalized MMSN-L1. In the case of MMSN-L2 it was, however, possible to see some loss of capacity.

## Conclusions

In summary, click chemistry such as thiol–ene or Huisgen's reaction was very efficient for the syntheses of cyclen-based ligands possessing triethoxysilyl group. Associated with microwaves, Huisgen's reaction showed successful preparation of challenging L3-*^t^*Bu ligand. L1-Boc and L2-*^t^*Bu ligands were grafted on magnetic mesoporous silica nanoparticles (MMSN) and protective groups were cleaved in order to extract and separate metal ions Ni(ii) and Co(ii) from model solutions. The kinetic of metal(ii) uptake was very quick reaching 80% of the capacity after 1–2 hours. The adsorption capacity of MMSN was very high, up to 2.13 mmol g^−1^ of Ni(ii) with ligand L1 grafted on MMSN.

## Conflicts of interest

There are no conflicts to declare.

## Supplementary Material

RA-011-D1RA01581H-s001
