# Supplementary material for: Synthesis of triethoxysilylated cyclen derivatives, grafting on magnetic mesoporous silica nanoparticles and application to metal ion adsorption
Source: RSC Adv. 2021 Mar 12;11(18):10777–84. doi: 10.1039/d1ra01581h (PMC8695893; doi:10.1039/d1ra01581h)
Supplement: RA-011-D1RA01581H-s001 [file RA-011-D1RA01581H-s001.pdf]

Electronic Supplementary Material (ESI) for RSC Advances.
This journal is © The Royal Society of Chemistry 2021

Electronic supplementary information (ESI)

Synthesis of triethoxysilylated cyclen derivatives, grafting on magnetic mesoporous silica nanoparticles and application to metal ion adsorption

Hao Li,[a,b] Mathilde Ménard,[a] Ani Vardanyan,[c] Clarence Charnay,[a] Laurence Raehm,[a] Erwan Oliviero,[a] Gulaim A. Seisenbaeva,[c] Roser Pleixats,[b] and Jean-Olivier Durand [a]

[a] ICGM, Univ. Montpellier, CNRS, ENSCM, Case 1701, Place Eugène Bataillon, CEDEX 05, 34095 Montpellier, France

[b] Department of Chemistry and Centro de Innovación en Química Avanzada (ORFEO-CINQA), Universitat Autònoma de Barcelona, 08193-Cerdanyola del Vallès, Barcelona, Spain.

[c] Department of Molecular Sciences, Swedish University of Agricultural Sciences, 750 07 Uppsala, Sweden.

1. General remarks

The ^1H and ^{13}C NMR spectra in solution were recorded on Bruker DPX-360 spectrometers, operating at ^1H NMR frequencies of 360.13 MHz, and at 298.0 K of temperature. All the spectra were calibrated using the residual solvent signal ($CHCl_3$, δ_H, 7.26 and δ_C, 77.16 ppm). Chemical shift data were expressed in ppm and coupling constant (*J*) values in Hz. Multiplicity of peaks was abbreviated as s (singlet), d (doublet), t (triplet), q (quartet) and dd (doublet of doublets). These NMR instruments belong to the *Servei de Ressonància Magnètica Nuclear* of the *Universitat Autònoma de Barcelona* (UAB). From the *Servei d'Anàlisi Química* of UAB the following experimental data were acquired: mass-spectrometry (MS) and elemental analysis (EA). Low- and high-resolution mass spectra were obtained by direct injection of the sample with electrospray techniques in a Hewlett-Packard 5989A and microTOF-Q instruments respectively. Elemental analysis of C, N and H were performed using Flash 2000 Organic Elemental analyser of Thermo Fisher Scientific with BBOT as an internal standard. The nitrogen-sorption measurements, FTIR, TEM and TGA analyses were performed at *Institut Charles Gerhardt Montpellier* (France). Dynamic light scattering (DLS) and Zeta potential (ζ) were obtained from the University of Montpellier. DLS analyses were performed using a Cordouan Technologies DL 135 particle size analyzer instrument. Zeta potential measurements were performed on MALVERN Instruments ZETA SIZER Nano series with 1-3 mg samples dispersed in 5 mL of aqueous solution (distilled water or 0.01 M NaCl solution) between pH 4 and 9. FTIR spectra were recorded in the 4000-400 cm^{-1} range using 32 scans at a nominal resolution of 4 cm^{-1} with a Perkin Elmer 100 FT spectrophotometer equipped with an ATR unit. TEM images were recorded with JEOL 1200 EXII microscope (JEOL Europe SAS, Croissy Sur Seine, France). HRTEM analyses were performed on a JEOL 2200 FS, operated at 200 kV. Images were recorded near Scherzer defocus on a 4kx4k CCD camera (Gatan US4000). BET was carried out with a 3Flex Version 5.00 apparatus, the sample was calcined at 600°C for 10 hours and degassed at 120°C for 10 hours. For the purpose of TEM analysis, the sample particles were dispersed in ethanol and then dropped onto copper grids covered with porous carbon films. TGA analyses were performed with a thermal analyser STA 409 Luxx® (Netzsch) in the range 25-800 °C by a heating speed of 5 °C/min. If necessary, experiments were carried out in Schlenk techniques with standard high vacuum. CTAB, NH_4NO_3, sodium hydroxide, potassium bromide and some reagents were purchased from Sigma-Aldrich.

Ethanol and acetone were purchased from Fisher Chemicals and hydrochloric acid from VWR PROLABO.

2. Synthesis of 4,7,10-tris(2-(*tert*-butoxy)-2-oxoethyl)-1,4,7,10-tetraazacyclododecan-1-ium bromide 3[1]

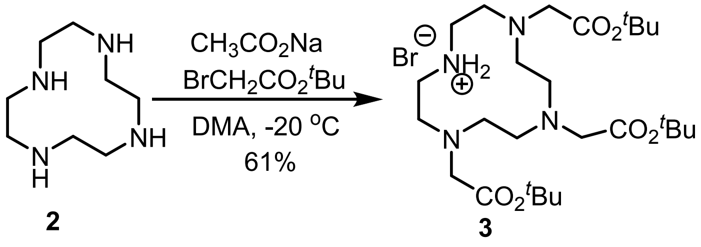

To a suspension of cyclen **2** (2.00 g, 11.62 mmol) and $CH_3CO_2Na \cdot 3H_2O$ (5.26 g, 38.4 mmol) in DMA (24 mL) at -20 °C was added dropwise a solution of *tert*-butyl bromoacetate (7.48 g, 38.4 mL, 96 mmol) in DMA (20 mL) over a period of 0.5 h. The reaction temperature was maintained at -20 °C during the addition, after which the reaction mixture was allowed to reach room temperature. After 24 h of vigorous stirring, the reaction mixture was poured into water (100 mL) to give a clear solution. Solid $NaHCO_3$ (5.05 g, 60.1 mmol) was added portion wise, the precipitate was collected by filtration and dissolved in $CHCl_3$ (80 mL). The solution was washed with water (60 mL), dried with anhydrous Na_2SO_4, filtered, and concentrated to about 10 mL. Diethyl ether was added, after which the final product **3** precipitated as a white solid, (4.2 g, 61% yield). **^{1}H NMR (360 MHz, $CDCl_3$) *δ* (ppm)**: 10.04 (br s, 2H), 3.37 (s, 4H), 3.29 (s, 2H), 3.09 (br s, 4H), 2.92-2.88 (m, 12H), 1.46 (s, 27H); **^{13}C NMR (91 MHz, $CDCl_3$) *δ* (ppm)**: 170.56, 81.66, 58.17, 51.30, 49.27, 47.52, 28.19.

3. Synthesis of tri-*tert*-butyl 2,2',2''-(10-allyl-1,4,7,10-tetraazacyclododecane-1,4,7-triyl)triacetate

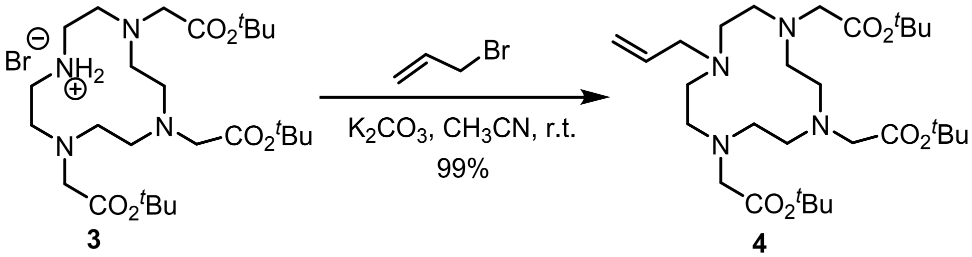

To a solution of hydrobromide **3** (2.51 g, 4.22 mmol) in acetonitrile (40 ml), potassium carbonate (1.46 g, 10.56 mmol) was added and the suspension was stirred for 30 min at room temperature. The mixture was placed in an ice-bath and allyl bromide (511.1 mg, 4.22 mmol) was added dropwise. The ice-bath was removed, and the reaction mixture was allowed to reach room temperature and stirred overnight at room temperature. The inorganic solid was removed by filtration, and the solvent evaporated under reduced pressure. The resulting oil was dissolved in hot toluene and filtered to remove the inorganic residue. After evaporation of the solvent under reduced pressure the product was obtained as a yellow oil (2.32 g, 99 %). **^{1}H NMR (360 MHz,** $CDCl_3$) δ (ppm): 5.91-5.83 (m, 1H), 5.18-5.09 (m, 2H), 3.28- 3.31 (m, 6H), 3.06 (d, J = 3.6 Hz, 2H), 2.83 (s, 12H), 2.62 (t, J = 7.2 Hz, 4H), 1.45 (s, 27H). **^{13}C NMR (91 MHz, $CDCl_3$) δ (ppm):** 171.2, 136.1, 117.2, 80.7, 59.0, 56.7, 56.3, 52.1, 51.9, 51.7, 28.2. **MS (ESI) m/z**: 387.2, 443.3, 499.3, 553.4, 555.4; **HRMS (ESI) m/z** $[M]^+$ calcd for $C_{29}H_{54}N_4O_6$: 554.4146, found: 554.4099.

4. Synthesis of tri-*tert*-butyl 2,2',2''-(10-(prop-2-yn-1-yl)-1,4,7,10-tetraazacyclododecane-1,4,7-triyl)triacetate 5[2]

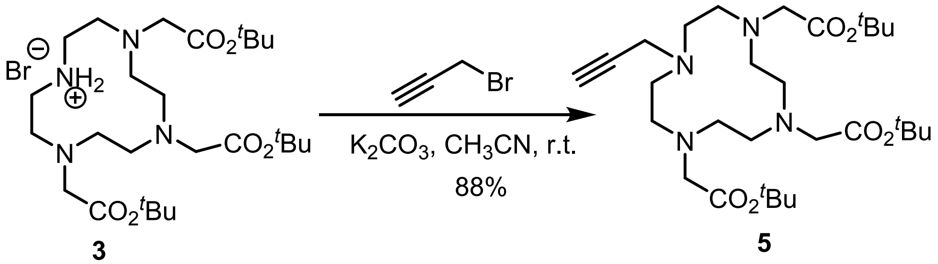

To a solution of hydrobromide **3** (4.19 g, 7.05 mmol) in acetonitrile (70 ml), was added potassium carbonate (1.95 g, 14.10 mmol) and the suspension was stirred for 30 min. The solution was placed in an ice-bath and propargyl bromide (80% in toluene, 1.05 g, 7.05 mmol) was added dropwise. The ice-bath was removed, the reaction mixture was allowed to reach room temperature and stirred overnight at room temperature. The inorganic solid was removed by filtration, and the solvent evaporated under reduced pressure. The resulting oil was dissolved in hot toluene and filtered to remove the inorganic residue. After evaporation of the solvent under reduced pressure the product **5** was obtained as a yellow oil (3.27 g, 88 %). **^{1}H NMR (360 MHz, $CDCl_3$) δ (ppm)**: 3.43 (d, J = 3.6 Hz, 2H), 3.28 (s, 6H), 2.82- 2.79 (m, 12H), 2.70- 2.67 (m, 4H), 2.14-2.13 (m, 1H), 1.45 (s,

27H); **^{13}C NMR (91 MHz, $CDCl_3$) *δ* (ppm)**: 171.12, 80.72, 79.31, 72.51, 56.78, 52.14, 51.80, 51.61, 43.08, 28.23.

5. ^{1}H NMR of the ligands

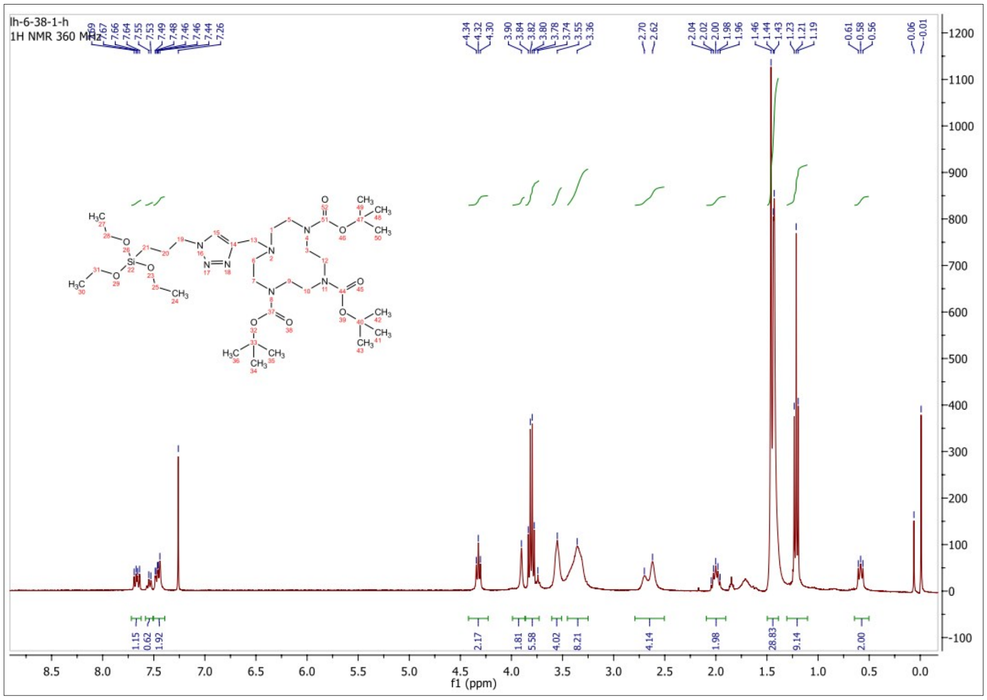

Figure S1. ^{1}H NMR of **L1-Boc**

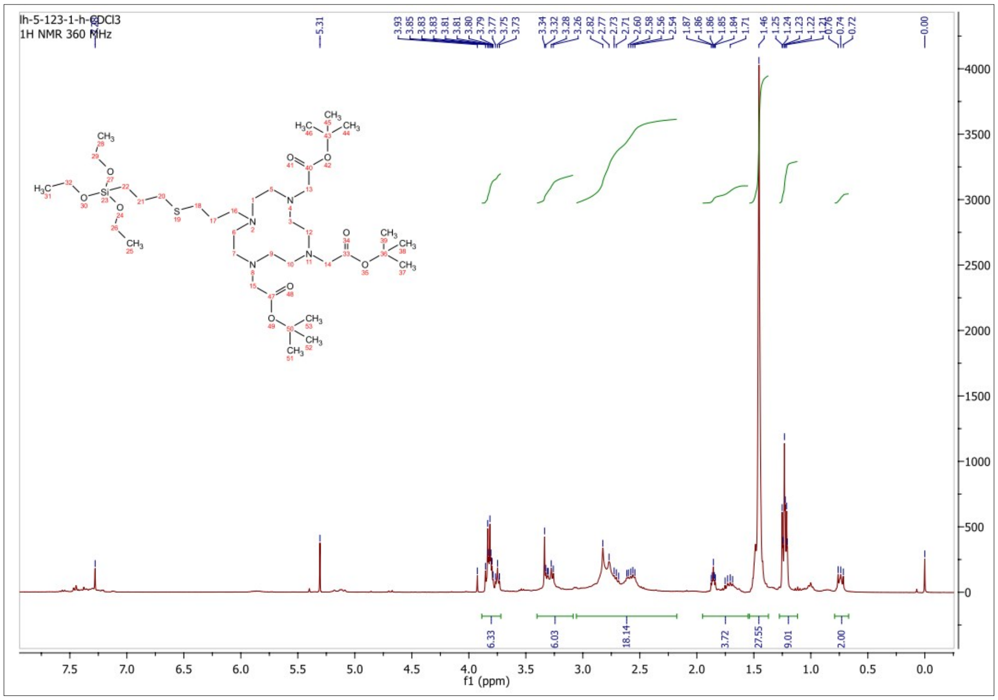

Figure S2. ^{1}H NMR of **L2-^{t}Bu**

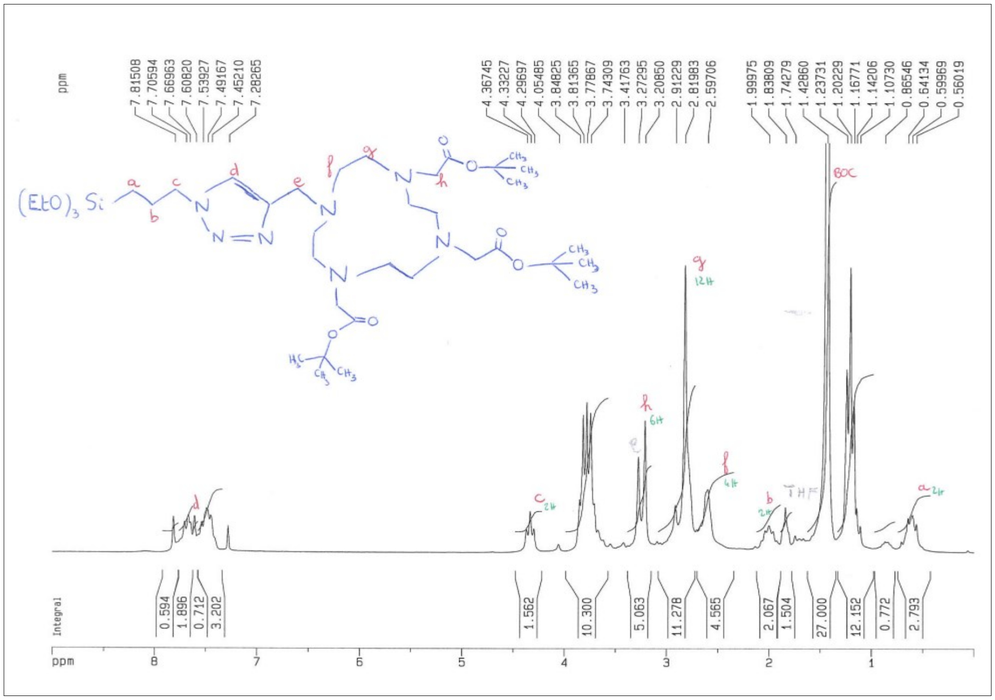

Figure S3-1. ^{1}H NMR of **L3-^{t}Bu**

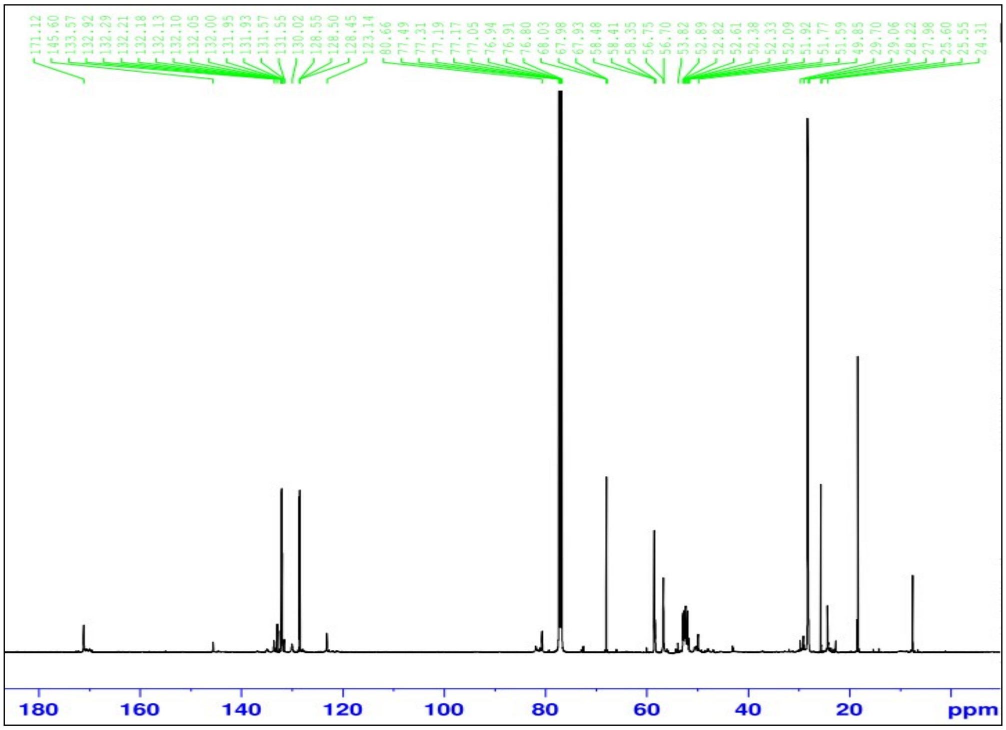

Figure S3-2. ^{13} C NMR of **L3-^{t}Bu**

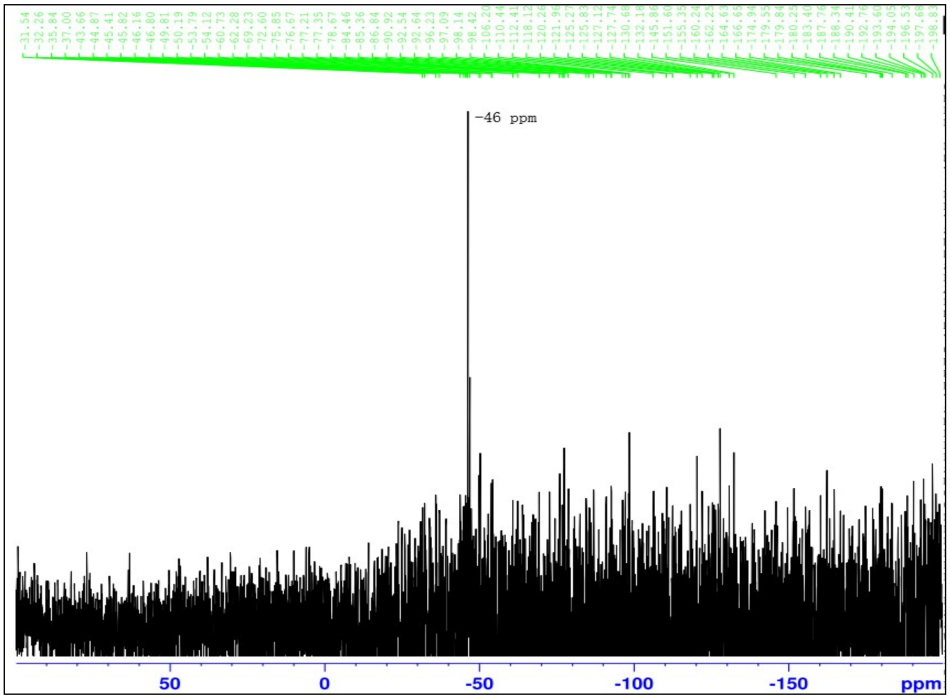

Figure S3-3. ^{29} Si NMR of **L3-^{t}Bu**

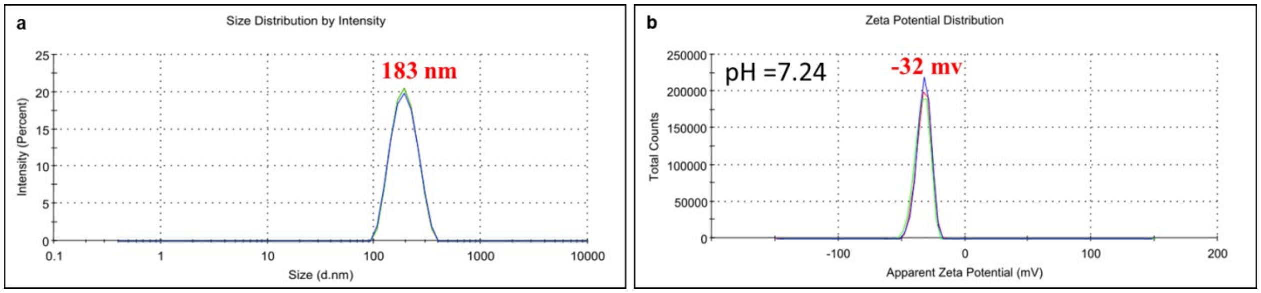

Figure S4. a) DLS size distribution of **MMSN** and b) Zeta potential of **MMSN**

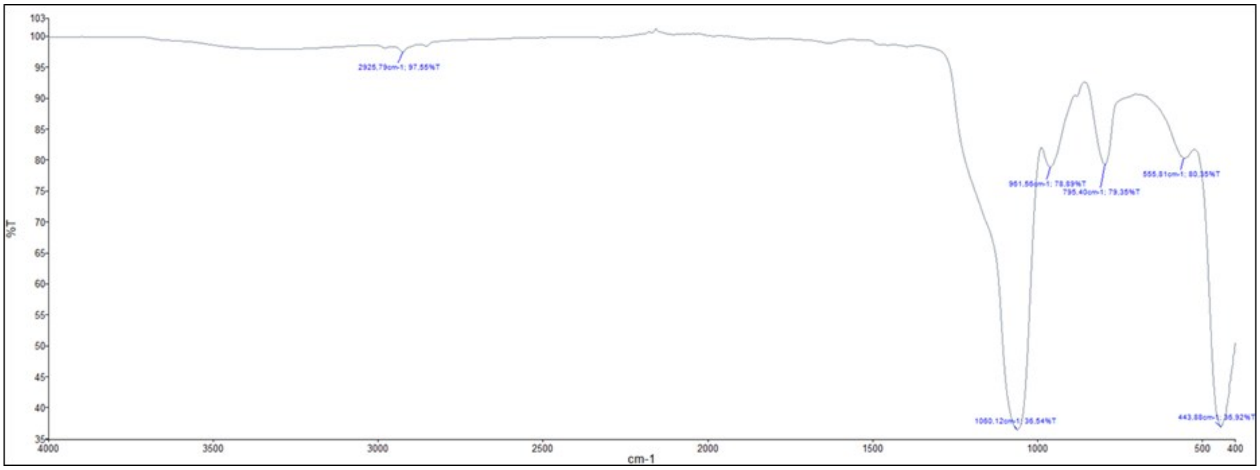

Figure S5. FTIR spectrum of **MMSN**

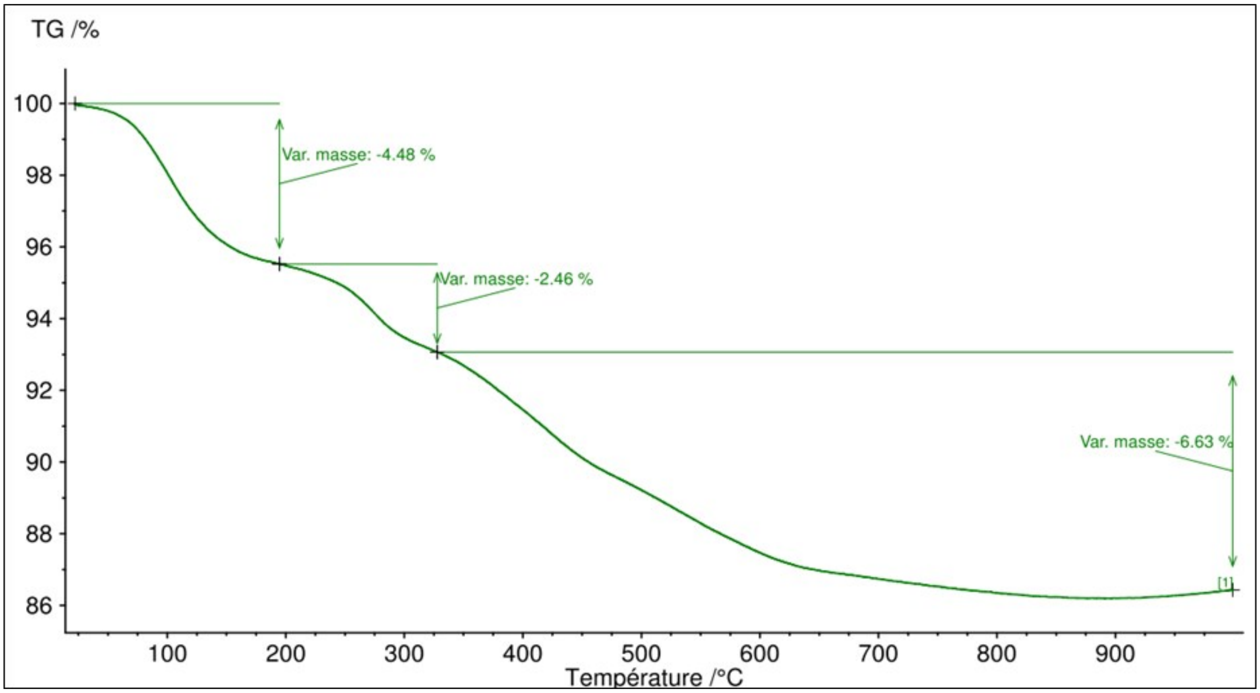

Figure S6. TGA of **MMSN**

Table S1. Investigation of surface grafting with ligand **L2**

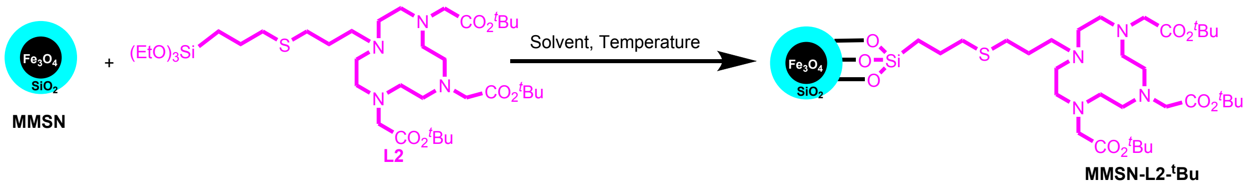

Entry	Reaction conditions [a]			γ(C=O) 1653-1657 cm^{-1} [c]	TGA[d]
	Solvent	Temperature (°C)	Ligand (mmol/g)[b]		
1	Toluene	135	1	×	nd[e]
2	DMF	90	1	√	18.1%
3	DMF	120	1	√	18.2%
4	DMF	130	1	√	17.7%
5 [f]	DMF	120	1	√	17.0%
6	DMF	120	2	√	20.8%

[a] Post-grafting conditions. [b] Ligand amount per g **MMSN**. [c] determined by FTIR. [d] Loss mass, TGA with a heating rate of 5 °C/min from 25 to 1000 °C. [e] Not determined. [f] Without H_2O as catalyst.

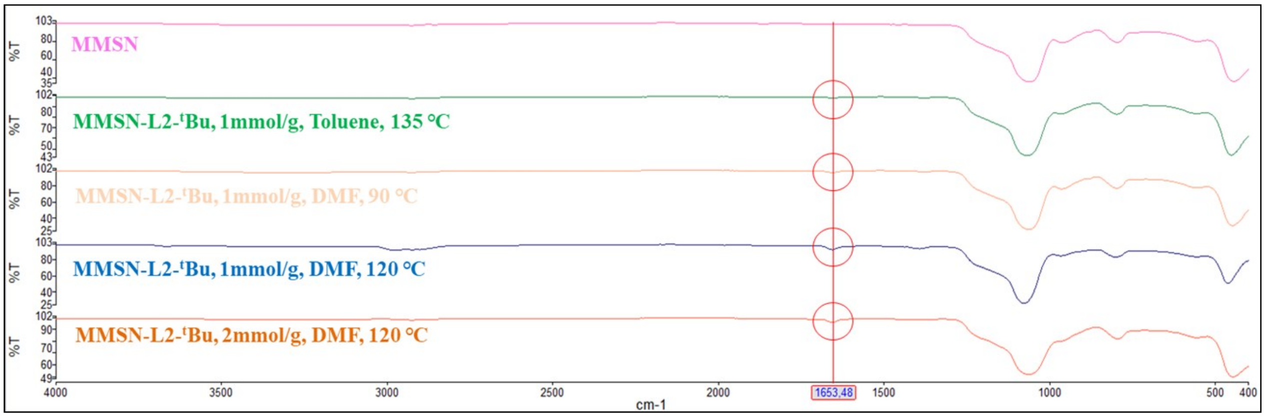

Figure S7. FTIR of **MMSN-L2-tBu** prepared in different conditions

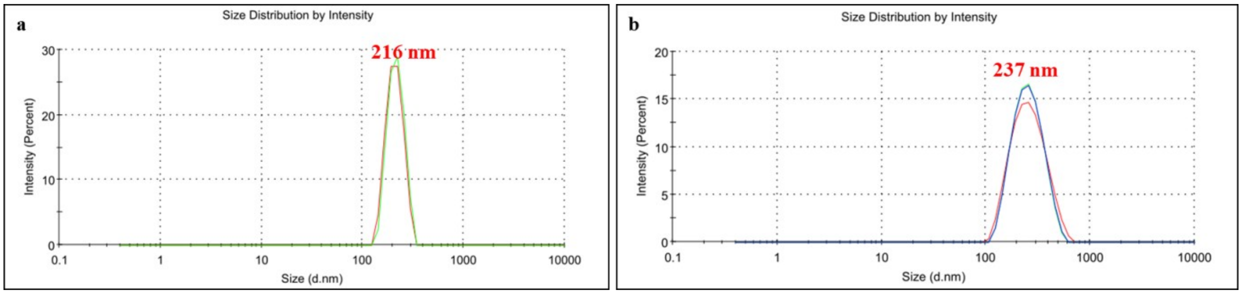

Figure S8. DLS of a) **MMSN-L1-Boc** and b) **MMSN-L2-^{t}Bu**

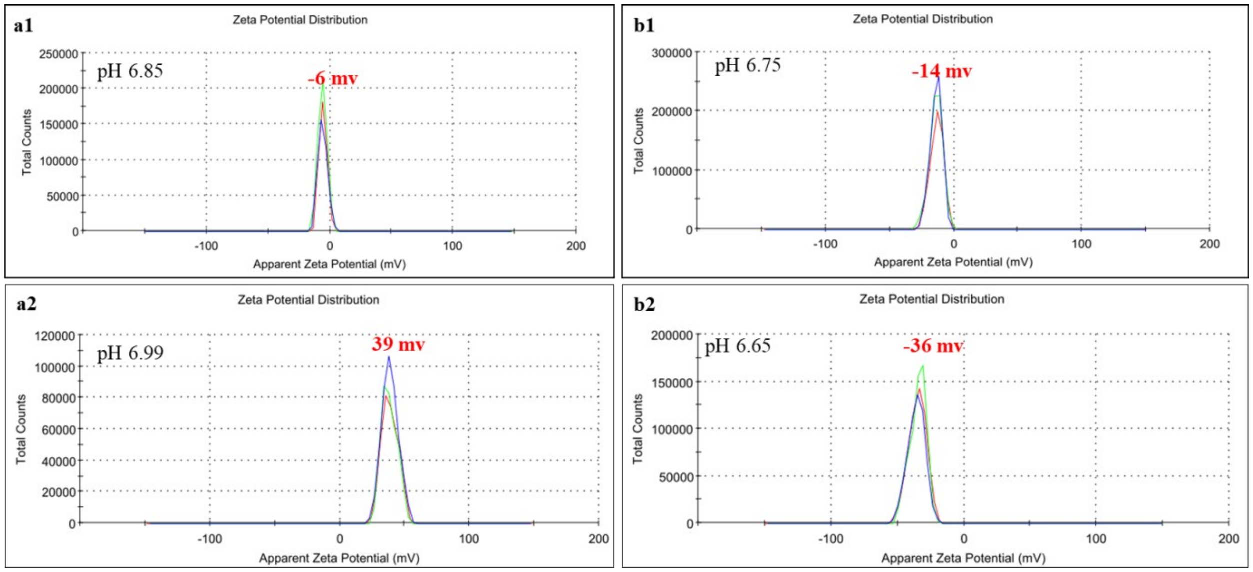

Figure S9. Zeta potential of a1) **MMSN-L1-Boc**, a2) **MMSN-L1** and b1) **MMSN-L2-^{t}Bu**, b2) **MMSN-L2**

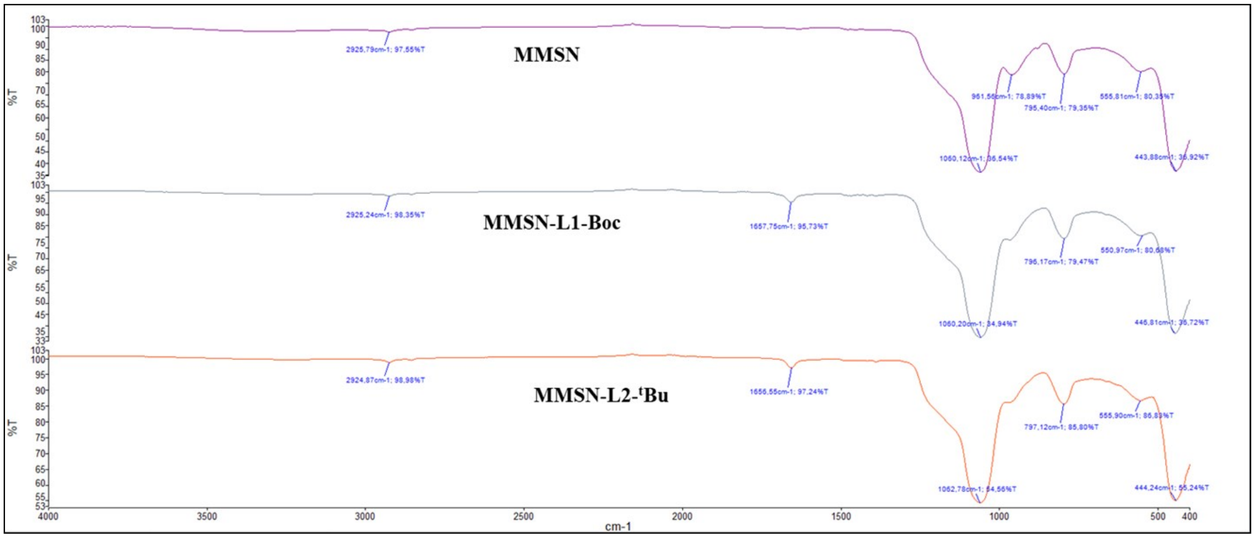

Figure S10. FTIR of **MMSN**, **MMSN-L1-Boc** and **MMSNs-L2-^{t}Bu**

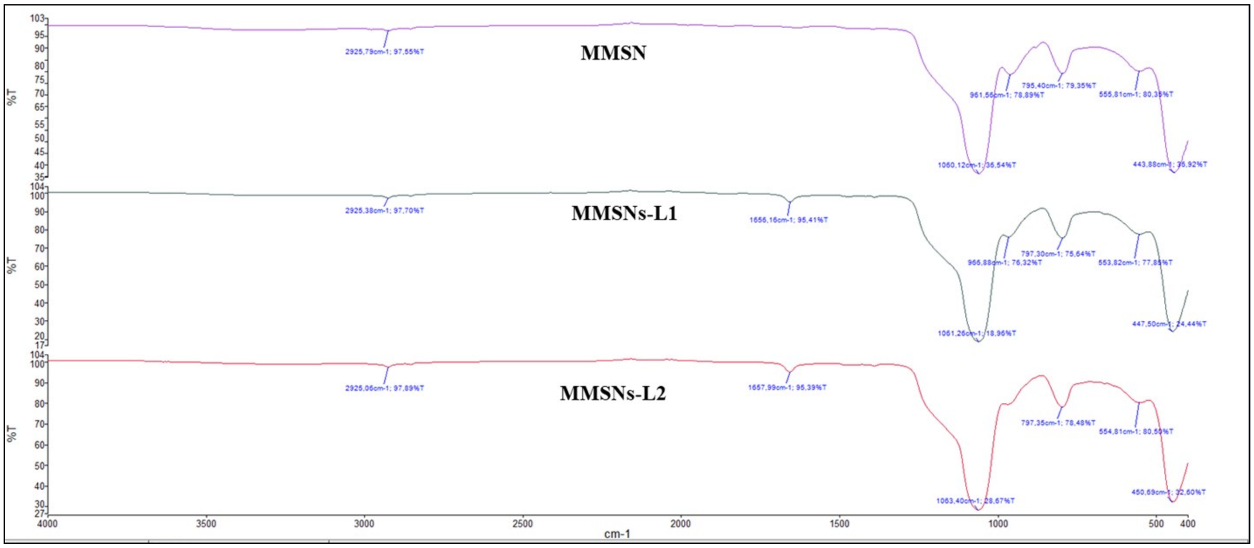

Figure S11. FTIR of **MMSN**, **MMSN-L1** and **MMSNs-L2**

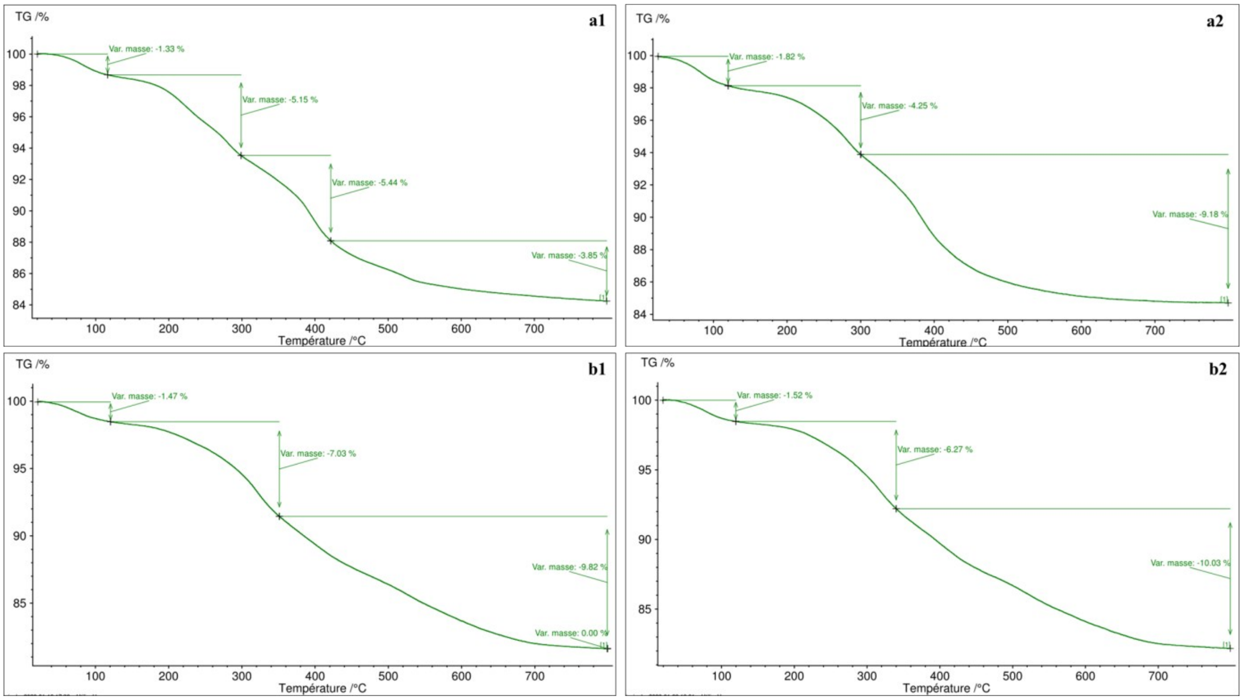

Figure S12. TGA analysis of a1) **MMSN-L1-Boc**, a2) **MMSN-L1** and b1) **MMSN-L2-^{t}Bu**, b2) **MMSN-L2**

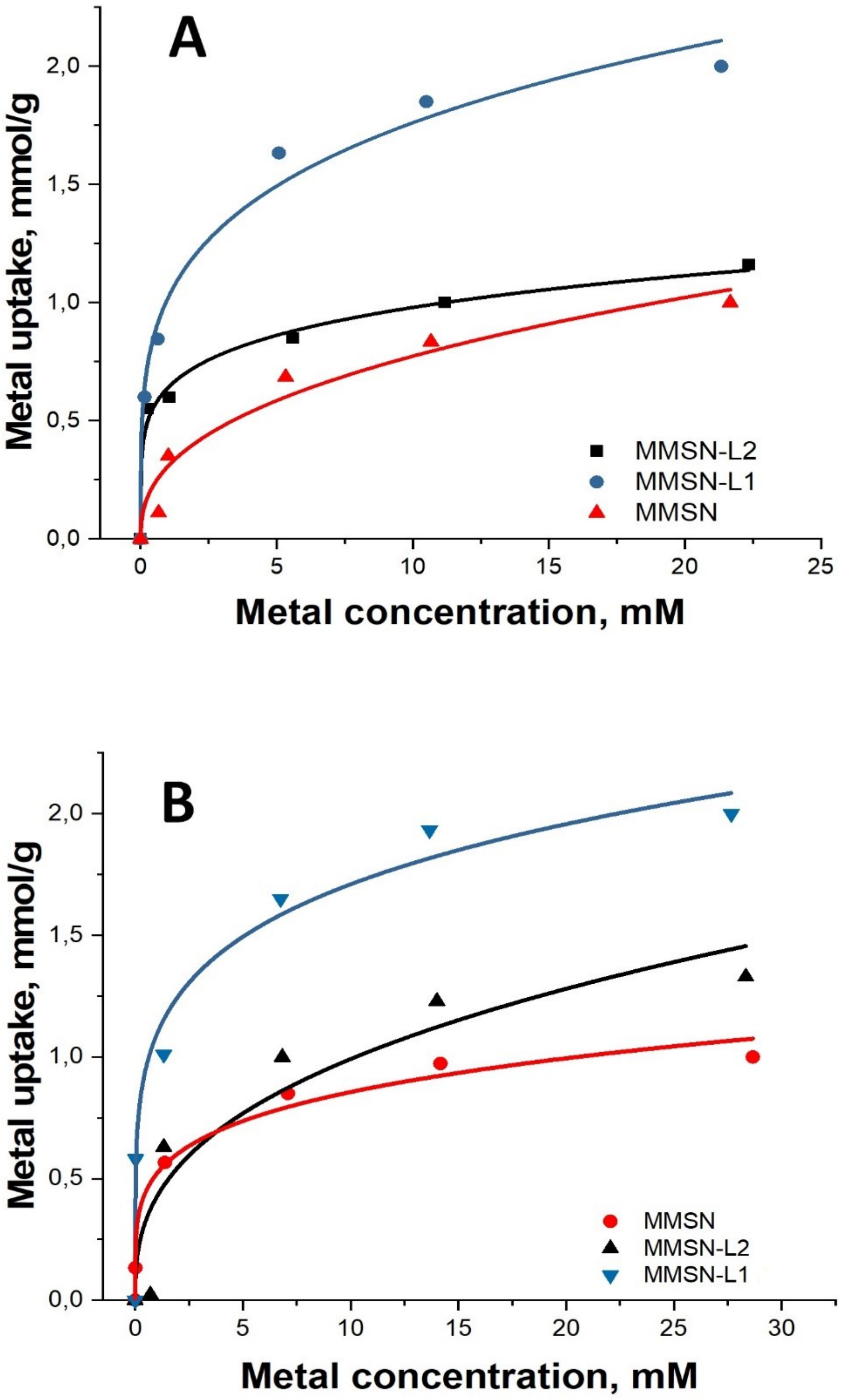

Figure S13. Freundlich isotherms for Ni^{2+} and Co^{2+}, respectively

Recycling of magnetic nanoparticles

The average amounts of adsorbed and desorbed metal ions are presented in the chart below.

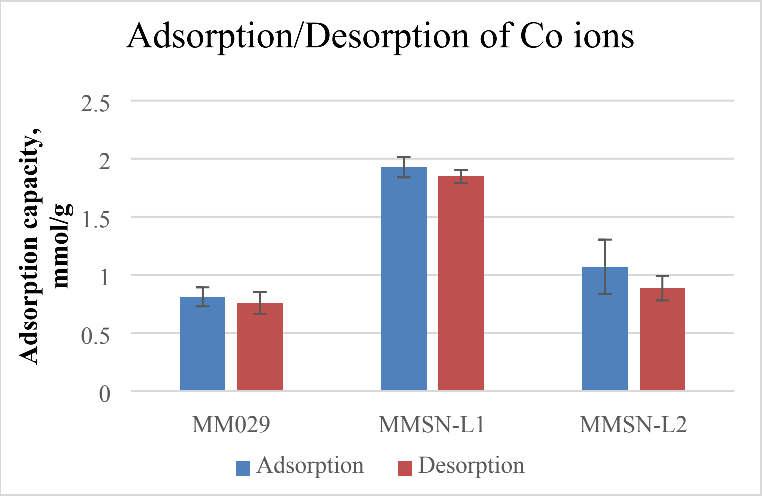

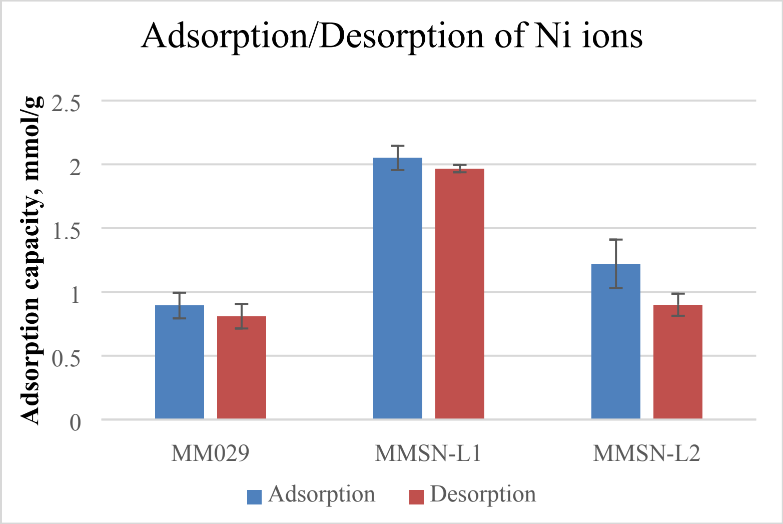

Figure S14. Cycling of the adsorbents for evaluation of reproducibility and stability.

Table S2. EDS analysis of MMSN for mixed Co and Ni ions

Map Sum Spectrum				
**Element**	**Line Type**	**Weight %**	**Weight % Sigma**	**Atomic %**
O	K series	49.29	0.48	55.48
Si	K series	31.35	0.32	20.10
C	K series	13.67	0.53	20.49
Fe	K series	3.42	0.16	1.10
N	K series	2.17	0.54	2.78

Co	K series	0.06	0.14	0.02
Ni	K series	0.05	0.15	0.02
Total		100.00		100.00

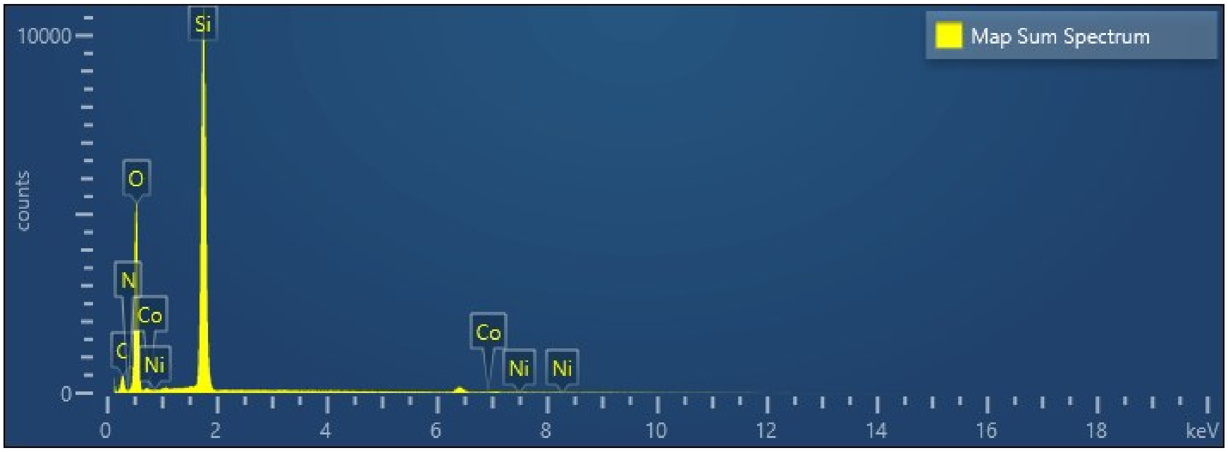

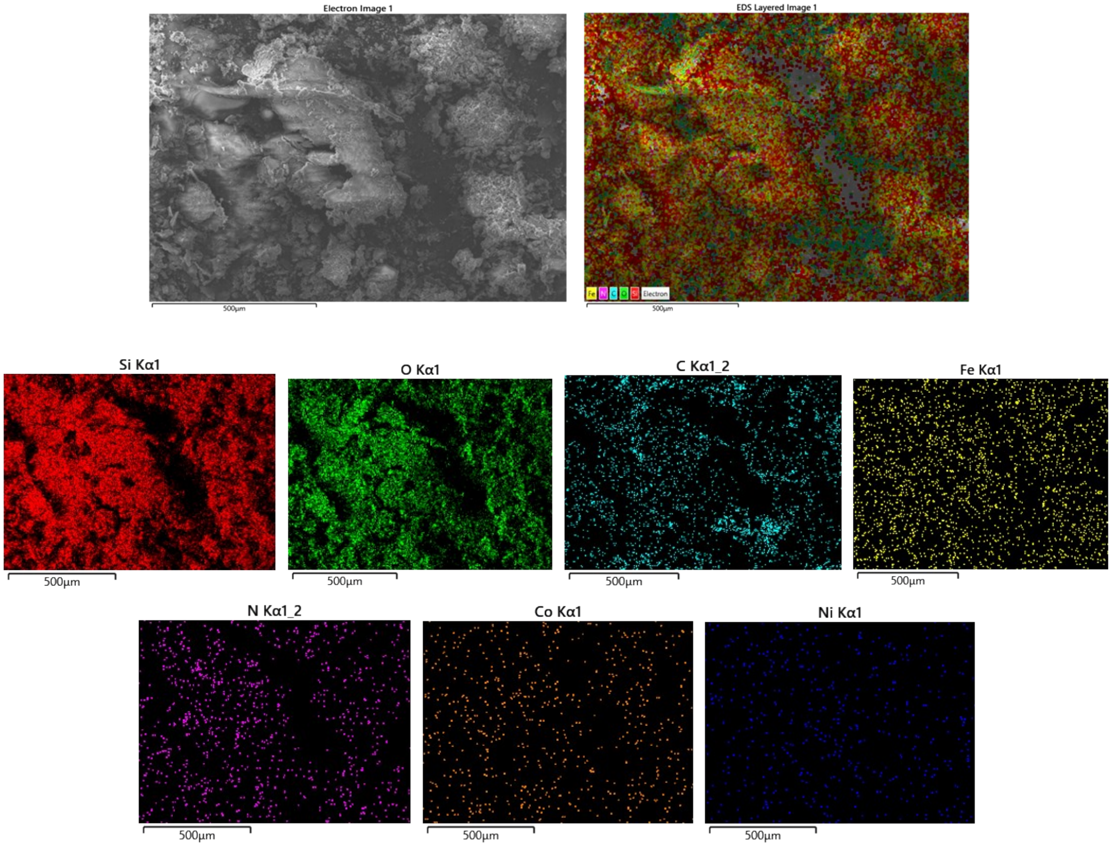

Table S3. EDS analysis of MMSN-L1 magnetic nanoparticles for mixed Co and Ni ions

Map Sum Spectrum				
**Element**	**Line Type**	**Weight %**	**Weight % Sigma**	**Atomic %**
O	K series	46.52	0.42	48.70
Si	K series	23.60	0.22	14.08
S	K series	1.18	0.04	0.62
C	K series	23.96	0.44	33.41
Fe	K series	2.42	0.11	0.73
N	K series	2.00	0.57	2.39
Co	K series	0.10	0.10	0.03
Ni	K series	0.21	0.11	0.06
Total		100.00		100.00

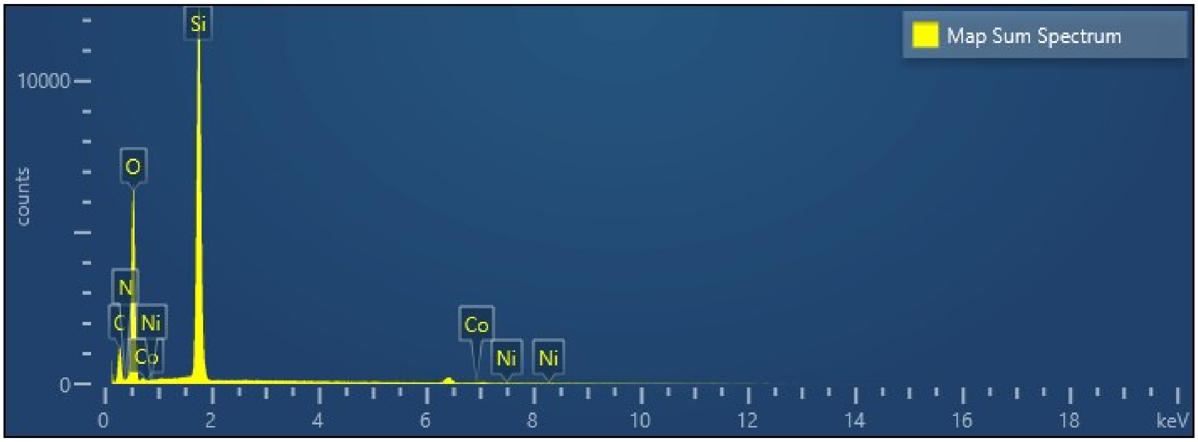

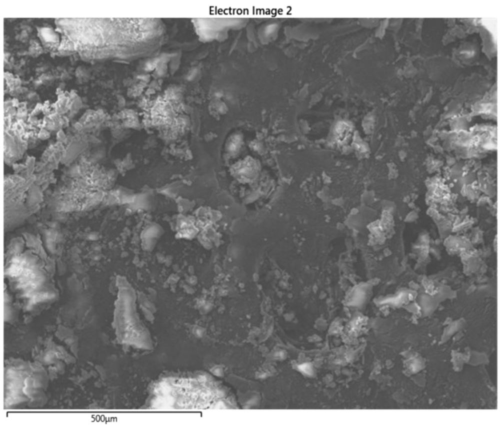

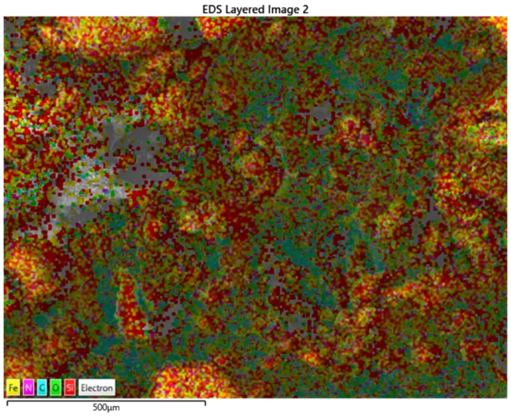

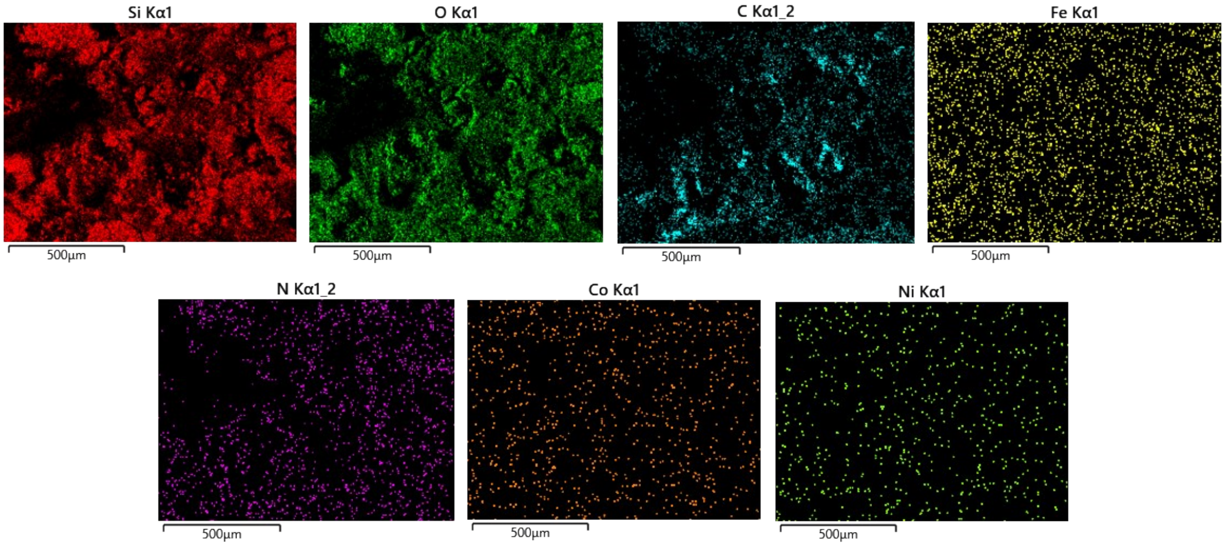

Table S4. EDS analysis of MMSN-L2 magnetic nanoparticles for mixed Co and Ni ions

Map Sum Spectrum				
**Element**	**Line Type**	**Weight %**	**Weight % Sigma**	**Atomic %**
O	K series	46.92	0.45	50.16
Si	K series	26.36	0.26	16.05
C	K series	21.68	0.48	30.88
Fe	K series	3.45	0.14	1.06
N	K series	1.49	0.60	1.82
Co	K series	0.05	0.12	0.01
Ni	K series	0.05	0.13	0.02
Total	100.00		100.00	

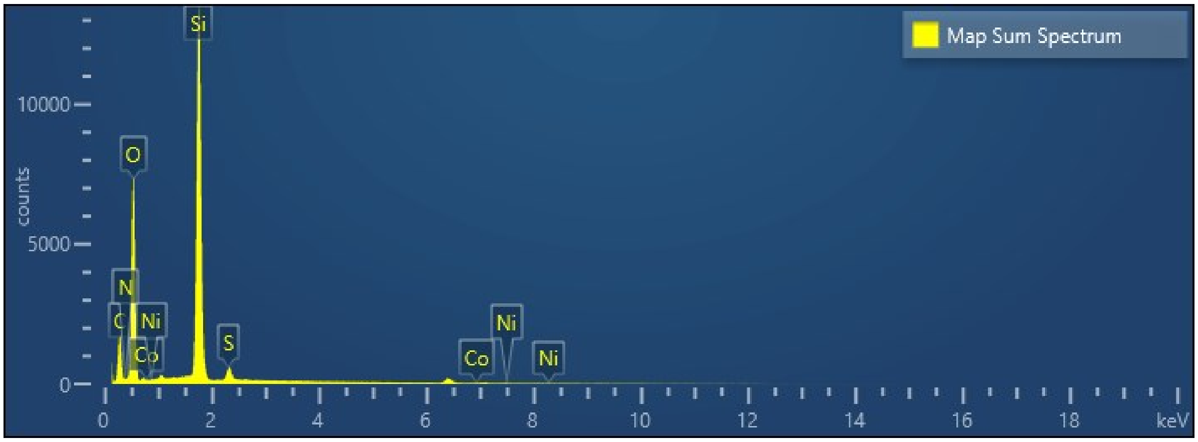

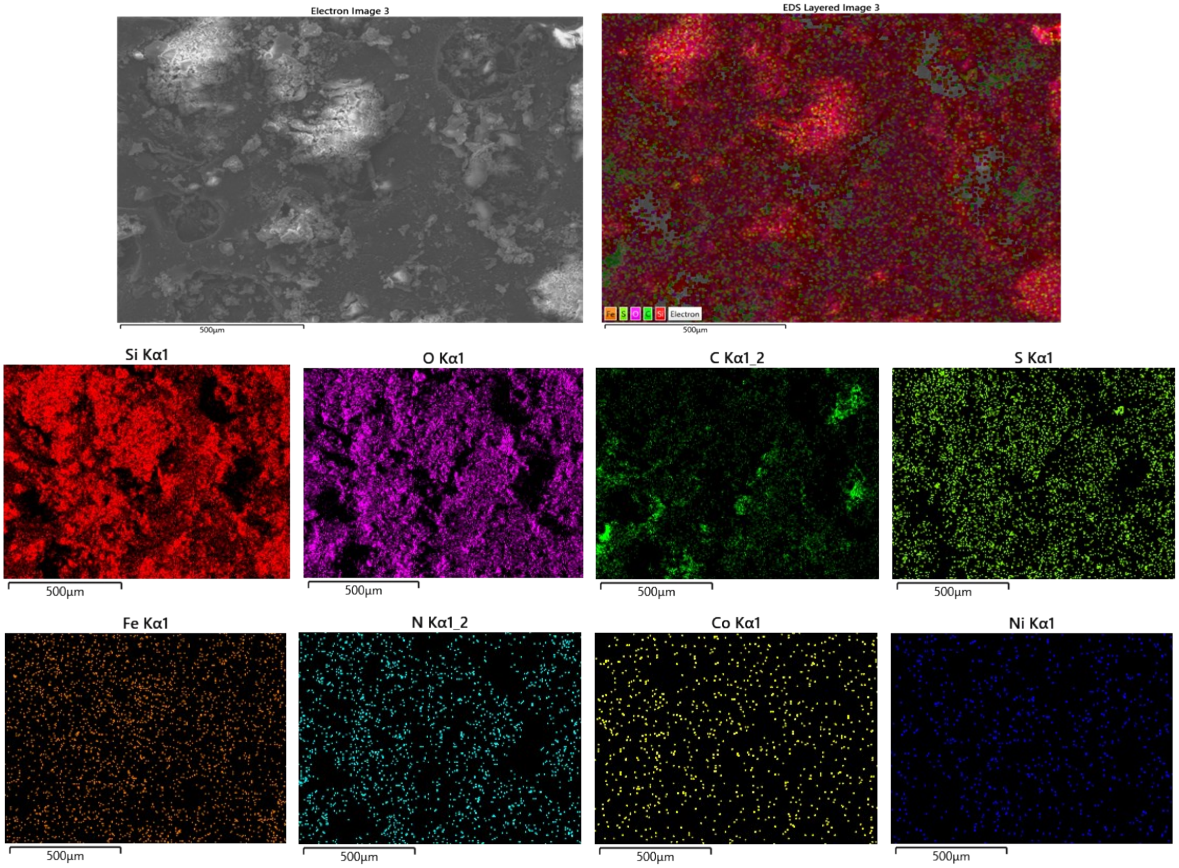

Table S5. Atomic % adsorption average (on 3 independent domains each) for MMSN and L1- and L2-grafted materials by EDS at x10000 magnification

	MMSN	MMSN-L1	MMSN-L2
Co	0.02	0.04	0.03

Ni	0.02	0.08	0.05

References

1. B. Jagadish, G. L. Brickert-Albrecht, G. S. Nichol, E. A. Mash, N. Raghunand, *Tetrahedron Lett.* **2011**, *52*, 2058.
2. A. K. R Junker, M. Tropiano, S. Faulkner, T. J. Sørensen, *Inorg. Chem.* **2016**, *55*, 12299.
